# Energy Landscape Reveals That the Budding Yeast Cell Cycle Is a Robust and Adaptive Multi-stage Process

**DOI:** 10.1371/journal.pcbi.1004156

**Published:** 2015-03-20

**Authors:** Cheng Lv, Xiaoguang Li, Fangting Li, Tiejun Li

**Affiliations:** 1 School of Physics, Peking University, Beijing, China; 2 LMAM and School of Mathematical Sciences, Peking University, Beijing, China; 3 Center of Quantitative Biology, Peking University, Beijing, China; Massachusetts Institute of Technology, United States of America

## Abstract

Quantitatively understanding the robustness, adaptivity and efficiency of cell cycle dynamics under the influence of noise is a fundamental but difficult question to answer for most eukaryotic organisms. Using a simplified budding yeast cell cycle model perturbed by intrinsic noise, we systematically explore these issues from an energy landscape point of view by constructing an energy landscape for the considered system based on large deviation theory. Analysis shows that the cell cycle trajectory is sharply confined by the ambient energy barrier, and the landscape along this trajectory exhibits a generally flat shape. We explain the evolution of the system on this flat path by incorporating its non-gradient nature. Furthermore, we illustrate how this global landscape changes in response to external signals, observing a nice transformation of the landscapes as the excitable system approaches a limit cycle system when nutrients are sufficient, as well as the formation of additional energy wells when the DNA replication checkpoint is activated. By taking into account the finite volume effect, we find additional pits along the flat cycle path in the landscape associated with the checkpoint mechanism of the cell cycle. The difference between the landscapes induced by intrinsic and extrinsic noise is also discussed. In our opinion, this meticulous structure of the energy landscape for our simplified model is of general interest to other cell cycle dynamics, and the proposed methods can be applied to study similar biological systems.

## Author Summary

Quantitatively understanding the dynamic behavior of the yeast cell cycle process under noise perturbations is a fundamental problem in theoretical biology. By constructing a global energy landscape for a simplified yeast cell-cycle regulatory network, we provide a systematic study of this issue. Our results demonstrate that the cell cycle trajectory is sharply confined as a canal bounded by ambient energy barriers, with the landscape adaptively reshaping itself in response to external signals, such as the nutrients improving and the activation of DNA replication checkpoint in our work. After performing quantitative analysis based on the landscape, We found that along the cell cycle trajectory, the typical width of the canal narrows and broadens periodically. Interestingly, this is also basically in accordance with the force strength of the dynamics. Additionally, in places where the driving force strength is comparable to the noise level, some additional pits form that are associated with the checkpoint mechanisms. Overall, our energy landscape study shows that the yeast cell cycle is a robust, adaptive and multi-stage dynamical process.

## Introduction

Stochasticity is an inherent property of living cells [1–6]. However, it is still difficult to quantify the robustness and adaptivity of cellular networks, even for a small cellular network perturbed by intrinsic random fluctuations, due to the massive cross regulations and nonlinear nature of such biological systems. As the size of the network grows, determining how to characterize the global stochastic dynamics of the system becomes a tough problem. “Waddington’s epigenetic landscape,” which utilizes potential energy to pictorially illustrate the dynamics and evolution of cellular networks, has been widely and repeatedly used for several decades [5, 7]. Some beautiful efforts and frameworks aiming to quantify this landscape have been made [8–13], but investigation into typical biological models still remains to be done. Furthermore, the energy landscape usually reshapes itself due to a variety of changes such as environmental signals [14], cell-cell interactions [15] and the growth rate dependence of protein concentrations [16]. Determining how to explicitly quantify this transformation for specific systems is also a major task.

The yeast cell cycle is an important biological process in which a cell reproduces itself through DNA replication and mitosis events, which are intimately related to the checkpoint mechanism [17, 18]. Recent work has revealed the dynamic regulatory mechanisms of the cell cycle, and the cell cycle process is now considered a series of irreversible transitions from one state to another [19–21]. The cell-cycle regulatory network must also be robust and adaptive to external stresses and signal changes. To quantitatively characterize this robustness, and provide a global description of the cell cycle regulatory system, some fundamental questions must be studied. For example, how does the energy landscape reflect the robustness and successive phases of the cell cycle? How does the landscape adaptively change in response to external signals? Is there any information that the energy landscape cannot provide? If so, does any other supplemental description exist?

Using a simplified budding yeast cell cycle model driven by intrinsic noise, we systematically explore the above issues from an energy landscape point of view by constructing a global quasi-potential energy landscape for the budding yeast cell cycle model. Our results demonstrate that the energy landscape of the cell cycle is globally attractive, and we show how the cell cycle regulatory network reduces fluctuations from its upstream process and enables long durations in the transition regime. We also describe how the landscape changes in response to external signals when nutrients become sufficient and the DNA replication checkpoint is activated. We also discuss the dynamic information provided by the non-gradient nature that the pure energy landscape cannot explain, and provide other approaches to take into account this non-gradient effect. In addition, we compare the difference between landscapes induced by intrinsic and extrinsic noise and discuss the finite volume effect. Overall, our energy landscape study shows that the budding yeast cell cycle is a robust, adaptive and multi-stage dynamical process.

## Methods

### Models

**Fig 1:**
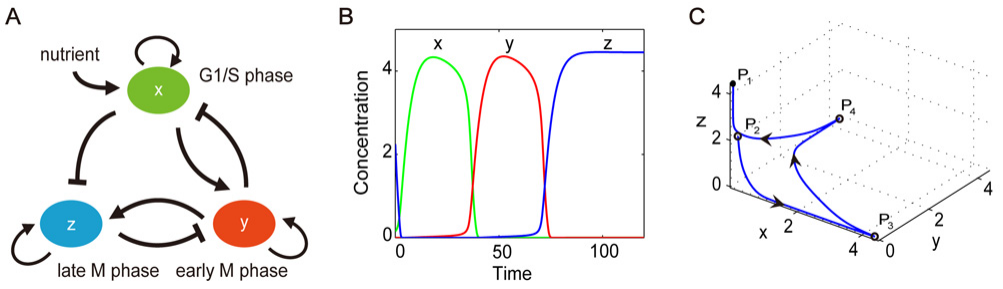
The model of the three-node yeast cell cycle network. (A) The network structure of the yeast cell cycle, where *x*, *y* and *z* represent key regulators of the G1/S, early M and late M modules, respectively. Different modules are connected by activation (lines end with arrow) and inhibition (line end with bar) interactions. (B) and (C) The evolution trajectory of the yeast cell cycle process with parameter values *j*_1_ = *j*_2_ = *j*_3_ = 0.5, *k*_1_ = *k*_2_ = *k*_3_ = 0.2, *k*_*i*_ = 5.0, *k*_*s*_ = 1.0, and *k*_*a*1_ = *k*_*a*2_ = 0.001. The system starts from *P*_2_ and evolves to *P*_1_. In (B), the time evolution of the variables *x*, *y* and *z* is shown with the green, red and blue lines, respectively.

We first assume that the DNA replication triggers the mitosis as a “domino” mechanism in the budding yeast cell cycle. That is, once the yeast cell passes the Start checkpoint, it will proceed through the whole cell cycle process spontaneously. Based on the key regulatory network [17] and our previous study on budding yeast [22], the cell cycle regulatory network can be simplified and separated into G1/S, early M and late M modules, as shown in Fig. 1A. We ignore the G2 phase for simplification. Each module has a positive feedback, and different modules are connected with activation and repression interactions. The deterministic equations describing this three-module yeast cell cycle network are

$$\begin{array}{cc} \frac{\;dx}{\;dt} & =\frac{x^{2}}{j_{1}^{2}+x^{2}}-k_{1}x-xy+a_{0}, \end{array}$$ (1a)

$$\begin{array}{cc} \frac{\;dy}{\;dt} & =\frac{y^{2}}{j_{2}^{2}+y^{2}}-k_{2}y-yz+k_{a1}x, \end{array}$$ (1b)

$$\begin{array}{cc} \frac{\;dz}{\;dt} & =\frac{k_{s}z^{2}}{j_{3}^{2}+z^{2}}-k_{3}z-k_{i}zx+k_{a2}y, \end{array}$$ (1c)

where *x* represents the concentrations of key regulators such as cyclins Cln1, 2, Clb5, 6 and transcriptional factors SBF and MBF in the excited G1 and S phases; *y* represents the concentrations of key regulators such as cyclins Clb1, 2 and transcriptional factor Mcm1/SFF in the early M phase; and *z* represents the concentrations of key inhibitors such as Cdh1, Cdc20 and Sic1 in the late M/G1 phase.

In this model, we assume simple forms to characterize the interactions. Thus, the first term in each equation, the second order Hill functions, represent the positive feedback in each module [23, 24]. The second term represents the degradation rate of each regulator, while the third term represents the repression or inhibition interaction between different modules. The parameter *a*_0_ in Equation (1a) characterizes the environmental nutrition condition [25, 26], and *k*_*a*1_ *x* and *k*_*a*2_ *y* are the trigger signals from *x* to *y* and *y* to *z* respectively. Starting from the excited G1 state, the system will finally evolve to a stable fixed point, the G1 state; if *a*_0_ is large enough, the system will enter the cell cycle process continually. With a proper parameter set, the model in Equation (1a) ensures a successive event order from DNA replication in the S phase to mitosis in the M phase, as well as a long duration for both events in the cell cycle process. For this work, we will simply denote the set of equations in Equation (1a) as d***x***/d*t* = ***b***(***x***), where ***x*** = (*x*, *y*, *z*).

The evolution trajectory in time and state space is shown in Fig. 1B and 1C, respectively. Here *P*_1_ (***x*** = (0, 0, *z*_max_)) represents the G1 state—which is the globally stable state of our model where *a*_0_ = 0.001—and *P*_2_ is a saddle point used to represent the excited G1 state from which the yeast cell passes the Start checkpoint and enters the cell cycle process. The trajectory in Fig. 1C can be separated into three parts. The first part is from the excited G1 (*P*_2_) to the S phase (*P*_3_), where ***x*** = (*x*_max_, 0, 0); the second part is from *P*_3_ to the early M state before the metaphase/anaphase transition (*P*_4_), where ***x*** = (0, *y*_max_, 0); and the third part evolves from *P*_4_ to the stable G1 state (*P*_1_). Compared with the model used in [9], our model does not rely on a quasi-steady-state assumption related to cell mass. More details about the network and model can be found in S1 Text and S1 Fig.

Starting from the deterministic descriptions above, we now address the stochastic setup of the system. The noise can be classified into the intrinsic and extrinsic types [1]. Here we model the intrinsic noise through a Gillespie jump process [27], in which the strength of the noise is determined by the reaction network structure. Denote the state of the system ***X*** = (*X*, *Y*, *Z*) where each component represents the number of molecules for the corresponding specie. We then translate each term in Equation (1a) into a chemical reaction. Taking Equation (1a) as an example, we have four associated reactions for the four terms. The state change vector ***ν*** for each reaction channel has the form ***ν***_1_ = ***ν***_4_ = [1, 0, 0] and ***ν***_2_ = ***ν***_3_ = [−1, 0, 0], which corresponds to the plus or minus sign in the equation. Once a reaction fires, the state of the system ***X*** would be updated to ***X***+***ν***. The reaction propensity function is determined by each term and the volume size (or system size) *V*, where *ϵ* ≡ *V* ^−1^ characterizes the magnitude of intrinsic fluctuations [28]. In Equation (1a), the four propensity functions are

$$\begin{array}{c} a_{1}\left(X\right)=\frac{VX^{2}}{{\left(j_{1}V\right)}^{2}+X^{2}},\;a_{2}\left(X\right)=k_{1}X,\;a_{3}\left(X\right)=\frac{XY}{V},\;a_{4}\left(X\right)=a_{0}V. \end{array}$$

We choose this form for the propensities because *a*(***X***) ∼ *O*(*V*) when ***X*** ∼ *O*(*V*), and the stochastic process ***x***(*t*) ≡ ***X***(*t*)/*V* will tend to the deterministic process Equation (1a) when the volume size *V* tends to infinity in this scaling. Equation (1b) and (1c) can be treated similarly. There are 12 reactions in total. The above setup is suitable for the intrinsic noise. For the extrinsic noise whose magnitude is independent of the considered system, we simply take the stochastic model as $\dot{x}=b(x)+\sqrt{\epsilon }\dot{w}$, where $\dot{w}$ is the standard temporal Gaussian white noise. More details can be referred to S1 Text.

### Algorithm

To study the robustness and adaptivity of our cell cycle model, we construct the Waddington-type energy landscape based on the concept quasi-potential from large deviation theory [29]. For any stable steady state ***x***_0_ of Equation (1a), the local quasi-potential *S*(***x***; ***x***_0_) with respect to ***x***_0_ is defined as

$$\begin{array}{c} S\left(x;x_{0}\right)=\underset{T>0}{\inf}\underset{\varphi \left(0\right)=x_{0},\varphi \left(T\right)=x}{\inf}\int_{0}^{T}L\left(\varphi ,\dot{\varphi }\right)dt, \end{array}$$ (2)

where inf is short for infimum, which means the least upper bound of a subset. *φ* is any connecting path and *L* is called the Lagrangian [29–31]. The concrete form of *L* is determined by the setup of the stochastic process defined through intrinsic or extrinsic fluctuations. In case that the driving noise is of white noise type, *L* can be obtained from path integral formulation [32]. *S*(***x***; ***x***_0_) tells us the difficulty of transition from state ***x***_0_ to ***x*** under the noise perturbation. The local quasi-potentials starting from different stable steady states can be suitably integrated together to form a global quasi-potential *S*(***x***), which is exactly our proposal to rationalize the Waddington landscape for any non-gradient system, i.e. the dynamic system whose driving force can not be simply represented by the gradient of a potential function [29, 31].

To better understand the quasi-potential, let us consider a special case. We suppose the dynamics is simply a gradient system with a single-well potential driven by small noise, i.e.

$$\begin{array}{c} \dot{x}=-\nabla U\left(x\right)+\sqrt{\epsilon }\dot{w}, \end{array}$$ (3)

where *ϵ* is a small parameter, and $\dot{w}$ is the standard temporal Gaussian white noise with $𝔼\dot{w}(t)=0$ and $𝔼\dot{w}(s)\dot{w}(t)=\delta (s-t)$. It is obvious that *U*(***x***) is one correct choice for the Waddington potential. We have the Lagrangian $L(\varphi ,\dot{\varphi })=|\dot{\varphi }+\nabla U(\varphi ){|}^{2}/2$ and *S*(***x***) = 2*U*(***x***) in this case, and the corresponding minimizing path satisfies the steepest ascent dynamics $\dot{\varphi }=\nabla U(\varphi )$ with the boundary condition *φ*(0) = ***x***_0_, *φ*(*T*) = ***x***, where ***x***_0_ is the unique potential energy minimum (see *SI* for details). This example shows that *S*(***x***) defined in Equation (2) gives the desired potential in the gradient case up to a multiplicative constant. It is also instructive to note that the quasi-potential

$$\begin{array}{c} S\left(x\right)=-\lim_{\epsilon \to 0}\epsilon \ln P\left(x\right), \end{array}$$ (4)

where *P*(***x***) ∝ exp(−2*U*(***x***)/*ϵ*) is the stationary Gibbs distribution of Equation (3). This result is also true for general dynamic systems [29].

For the Gillespie jump processes, there is no explicit form of *S*(***x***). However, the invariant distribution *P*(***x***) satisfies the chemical master equation

$$\begin{array}{c} \sum_{j}\left[a_{j}\left(x-\frac{\nu_{j}}{V}\right)P\left(x-\frac{\nu_{j}}{V}\right)-a_{j}\left(x\right)P\left(x\right)\right]=0 \end{array}$$ (5)

and we can plug the WKB ansatz [33] *P*(***x***) ∝ exp(−*VS*(***x***)) into Equation (5). Here the system size *V* plays the role of 1/*ϵ*, and this ansatz originates from Equation (4) essentially. The leading order term yields a Hamilton-Jacobi equation

$$\begin{array}{c} H(x,\nabla S(x))=0, \end{array}$$ (6)

where the Hamiltonian has the form

$$\begin{array}{c} H\left(x,p\right)=\sum_{j}a_{j}\left(x\right)\left(e^{p\cdot \nu_{j}}-1\right) \end{array}$$

for the standard Gillespie jump process. From classical mechanics, the *S*(***x***) obtained from WKB ansatz is exactly the quasi-potential defined through Equation (2). So even in the non-gradient case, we can still define *S*(***x***) as a generalization of the potential. That is why it is called quasi-potential. *S*(***x***) is also a Lyapunov function of the original deterministic system [34] Equation (1a).

The quasi-potential inherits key properties as the real potential guarantees in a gradient system. Besides logarithmically equivalent to the invariant distribution *S*(***x***) ∼ −*ϵ* ln *P*(***x***), the mean exit time *τ* that the system escapes from an attractive basin has the asymptotic form *τ* ∝ exp(*V*Δ*S*), where Δ*S* is the energy barrier height between the boundary of the basin and the stable state. The deeper the quasi-potential well is, the harder the system leaves a stable state. So the quasi-potential energy landscape describes the robustness of a system. This is similar for the extrinsic noise. For more properties about the quasi-potential, one may refer to [29–31] and S1 Text. In later text we will call *S*(***x***) the potential energy for simplicity.

The computation of *S*(***x***) by solving the equation *H*(***x***, ∇*S*) = 0 is not straightforward although there are already powerful methods [35, 36]. Since we are interested in both the energy landscape and the transition path, we choose to compute the energy landscape through the gMAM method [31, 37]. The idea is to directly minimize the action functional Equation (2) through Maupertuis principle for the space of curves (See *SI* for details). In our work, the constructed energy landscape *S*(***x***) is a function of three variables. For convenience of visualization and analysis, we plot *S*(***x***) in two dimensional planes with a certain dimension fixed. Therefore the global landscape is cut into different slices from various directions.

## Results

### Energy landscape of the yeast cell cycle network

Using the described method, we constructed the energy landscape *S*(***x***) for a budding yeast cell cycle network. We will state our findings from the energy landscape along the evolving path, i.e., from the excited G1 state (*P*_2_) to the final steady G1 state (*P*_1_).

**Fig 2:**
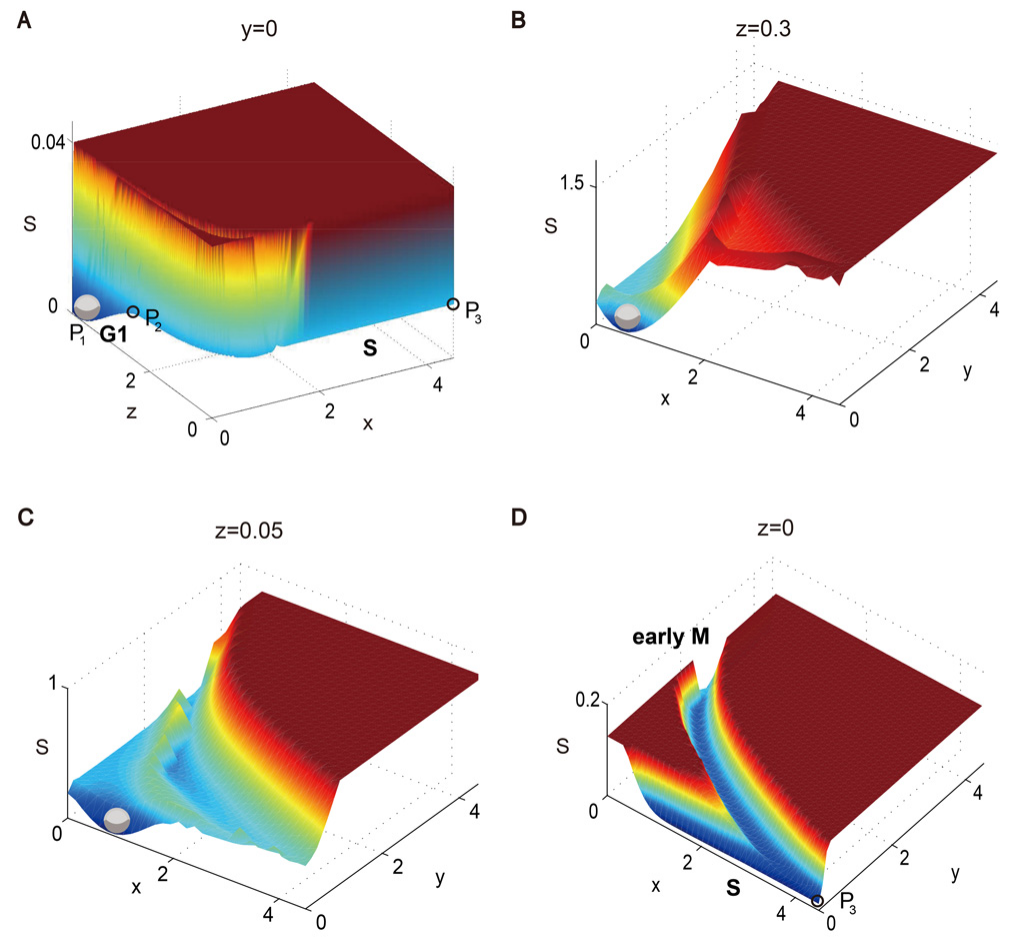
Different slices of the global energy landscape of the three-variable yeast cell cycle model. (A) The landscape on the *x*-*z* plane with *y* = 0 corresponds to the G1/S phase in the cell cycle process. (B) and (C) The landscapes on the *x*-*y* plane with *z* = 0.3 and *z* = 0.05. (D) The landscape on the *x*-*y* plane with *z* = 0 corresponds to the S phase and the early M phase transition. The “G1”, “S” and “early M” in bold refer to G1 phase, S phase and early M phase respectively.

We first focus on the section from *P*_2_ to *P*_3_. Fig. 2A illustrates the slice of the energy landscape on the *x*-*z* plane where *y* = 0. We can see that the G1 state is the global minimum on the energy landscape, and there exists an energy barrier between *P*_1_ and *P*_2_ to prevent small noise activation. Outside this potential well, the energy function *S*(***x***) along the first part of the trajectory (from *P*_2_ to *P*_3_) is relatively flat, while the energy cost to deviate from the cycling path is high. Intuitively, we will call the cell cycle trajectory a “canal” to illustrate its flatness along the path.

At the end of the first part of the cell cycle, the G1/S phase variable *x* gradually increases and represses *z* to zero. The S phase canal is quite narrow when it evolves near the vertex *P*_3_. As the system evolves through *P*_3_, *x* gradually triggers the activation of the early M phase variable *y*, at which point the activated *y* begins to repress *x*. This corresponds to the S/M transition of the yeast cell cycle and we denote it as the early M phase canal. The landscape of the S phase and S/M transition is illustrated in Fig. 2D where *z* = 0. In the bottom right corner of Fig. 2D, the energy barrier between the canals of the S and early M phases greatly decreases the probability that the system passes the S/M transition without crossing *P*_3_, hence ensuring the robustness of the S/M transition.

More details about the formation of the early M canal and the S/M transition are shown in Fig. 2B (the *z* = 0.3 plane) and 2C (the *z* = 0.05 plane). In Fig. 2B, the system is shown to be temporarily restricted to a small area on the *x*-*y* plane, isolated by barriers separating it from the G1 state and the early M canal. As *z* gradually decreases to 0.05, Fig. 2C shows that this restricting area is still small but slowly shifts to a place with a larger *x* value. In addition, the early M canal looks more apparent. When *z* finally falls to zero (Fig. 2D), the energy barrier between the S canal and the early M canal disappears. Now the cell can execute DNA replication events with a long duration across *P*_3_, after which it successively evolves to the M phase.

In the second part of the cell cycle, the system evolves through the early M flat canal to the vertex *P*_4_ over a sufficient duration for the mitosis event. When the system passes through *P*_4_, *y* triggers the activation of the late M variable *z*, and the activated *z* begins to repress *y*. This is the transition from the early M phase to the late M phase, corresponding to the metaphase/anaphase transition in yeast cell cycle process. The landscape on the *x* = 0 plane looks very similar with the one on the *z* = 0 plane (Fig. 2D) and is shown in S6 Fig. Finally, in the third part of the cell cycle, the activated *z* represses *y* to zero and the system evolves to a G1 stable state (*P*_1_) and waits for another cell cycle division signal.

### Non-gradient force and pseudo energy landscape

Since the main canal along the cell cycle trajectory in Equation (1a) after *P*_2_ is flat, the above energy landscape itself cannot tell us the moving direction of the system on the canal, which impels us to investigate its non-gradient nature. From large deviation theory, we know that the most probable cycling path under Gillespie’s stochastic jump dynamics satisfies

$$\begin{array}{c} \frac{dx}{dt}=\nabla_{p}H\left(x,\nabla S\right)=F\left(x\right), \end{array}$$ (7)

where *S*(***x***) is the energy landscape under discussion, ***p*** ≡ ∇*S*, and *H* is the Hamiltonian of the considered system. In general, ***F***(***x***) is not parallel to ∇*S*, and so we have additional non-gradient effects for the transition paths. This point is also emphasized in [8, 9]. However, if the gradient force becomes zero, we have ***F***(***x***) = ***b***(***x***), which exactly corresponds to the dynamical driving force in the deterministic Equation (1a) (See S1 Text for details). This is the case for the flat landscape along the canal in our results.

**Fig 3:**
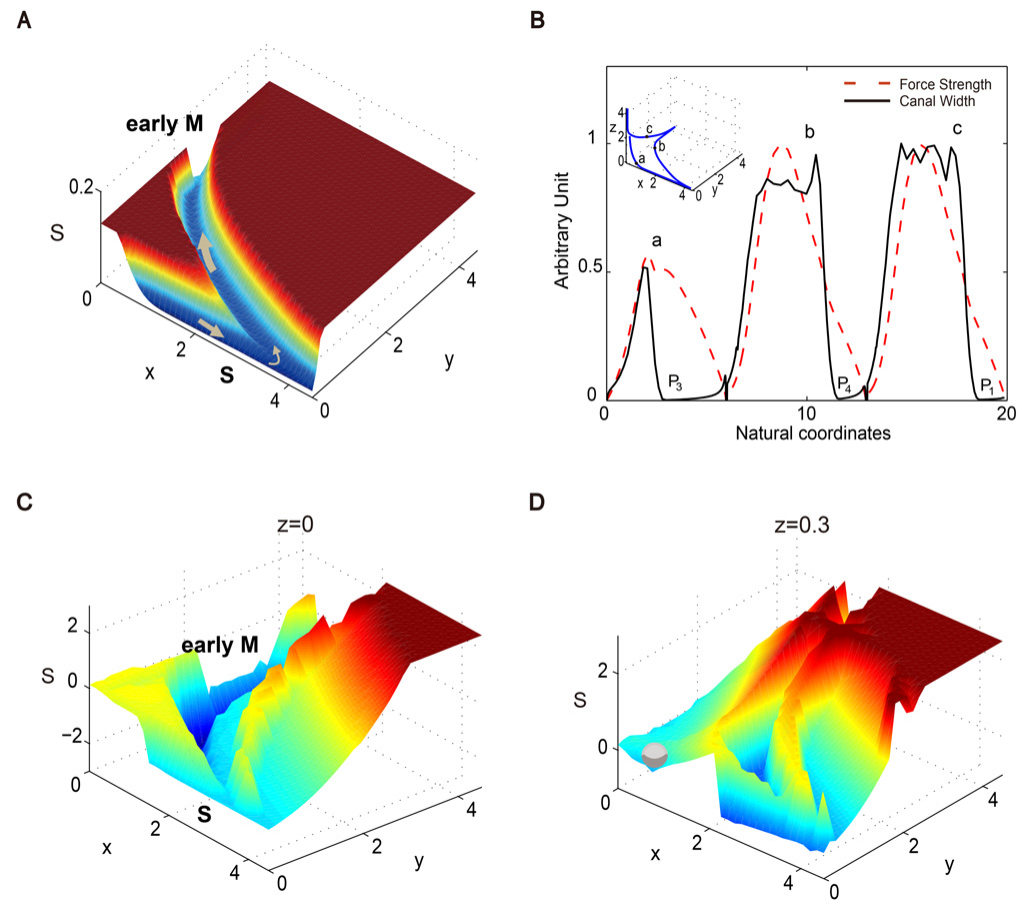
The effects of the non-gradient force in the yeast cell cycle trajectory. (A) The energy landscape with the force strength (gray arrows) on the *x*-*y* plane with *z* = 0. The length of the arrow is positively related to the force strength. (B) The driving force strength (red dashed line) and the fluctuation strength in the vertical direction (black line) along the cell cycle trajectory. For the *x*-axis, we use the natural coordinates of the cell cycle trajectory, i.e. the evolution distance from the start point *P*_2_. Inset: the evolution trajectory of the yeast cell cycle process where the letters “a”, “b” and “c” mark three points on the ODE trajectory with a large force strength. (C) and (D) The pseudo energy landscape on the *x*-*y* plane with *z* = 0 (C) and *z* = 0.3 (D). The “S” and “early M” in bold refer to S phase and early M phase respectively.

In Fig. 3A, we illustrate the force strength of ***F***(***x***) on the energy landscape in the *z* = 0 plane as an example. Along the cell cycle trajectory, we observe that the force strength *F*(***x***) is large in both S phase and early M phase canals, but extremely small near *P*_3_ and *P*_4_. Furthermore, we find that this tendency basically corresponds to the restriction width of the canal in the transverse direction (Fig. 3B). This means that, in the S phase and early M phase canals where the driving force is large, the cell cycle process will progress quite quickly, and hence does not require a strong restriction. As the process passes through *P*_3_ and *P*_4_, however, the system evolves more slowly (i.e. with more duration), and the driving force decreases and the canal width narrows in order to restrict fluctuations in the transverse direction. Thus, the dynamic properties around *P*_3_ work as an analogous DNA replication checkpoint. Similarly, the same characteristics around point *P*_4_ act as an analogous M phase checkpoint. Therefore, we suggest that this fast-slow dynamic and corresponding wide-narrow geometry of the landscape act as the dynamical mechanism keeping the cell cycle robust, precise and efficient.

To further visualize the non-gradient force in the flat canal, we came up with an alternate way of constructing the energy landscape, which we will refer to as the local pseudo energy landscape. The main idea behind this approach is to temporarily remove the globally stable state *P*_1_ from our model and only focus on the downhill flat canal after the saddle point *P*_2_. Thus the pseudo energy landscape is only a local landscape and no longer reflects the global stationary probability distribution (See S1 Text for details). In Fig. 3C and 3D we illustrate the pseudo energy landscape constructed using this method. The result is a bit like combining the original landscape and the non-gradient effect together. From Fig. 3C, we can see that the effect of the force strength is replaced by the steepness of the canal in the tangential direction, while the landscape in the transverse direction remains the same. We emphasize that this point is essential to explain the directionality of the dynamic path along the flat canal, where we have observed a phenomenological fact that the gradient of the global potential *S*(***x***) becomes zero and the driving force ***b***(***x***) gives the moving direction. In Fig. 3D we also show that the pseudo landscape on the *z* = 0.3 plane. Compared with Fig. 2B, we can see that the appearance of the S and early M canals is more apparent in the pseudo landscape. Furthermore, there exists a barrier between the S and early M canals (in the bottom right corner of Fig. 3D), which further guarantees the complete disappearance of the specie *z* before the cell enters the M phase.

### Energy landscape adaptively responds to external signals

In the previous results, we obtained and analyzed the energy landscape of the yeast cell cycle model in Equation (1a) using *a*_0_ = 0.001, *k*_*a*1_ = 0.001, *k*_*a*2_ = 0.001, and so on. However, when the external signal or stress changes, how does the energy landscape adaptively make such a transformation?

**Fig 4:**
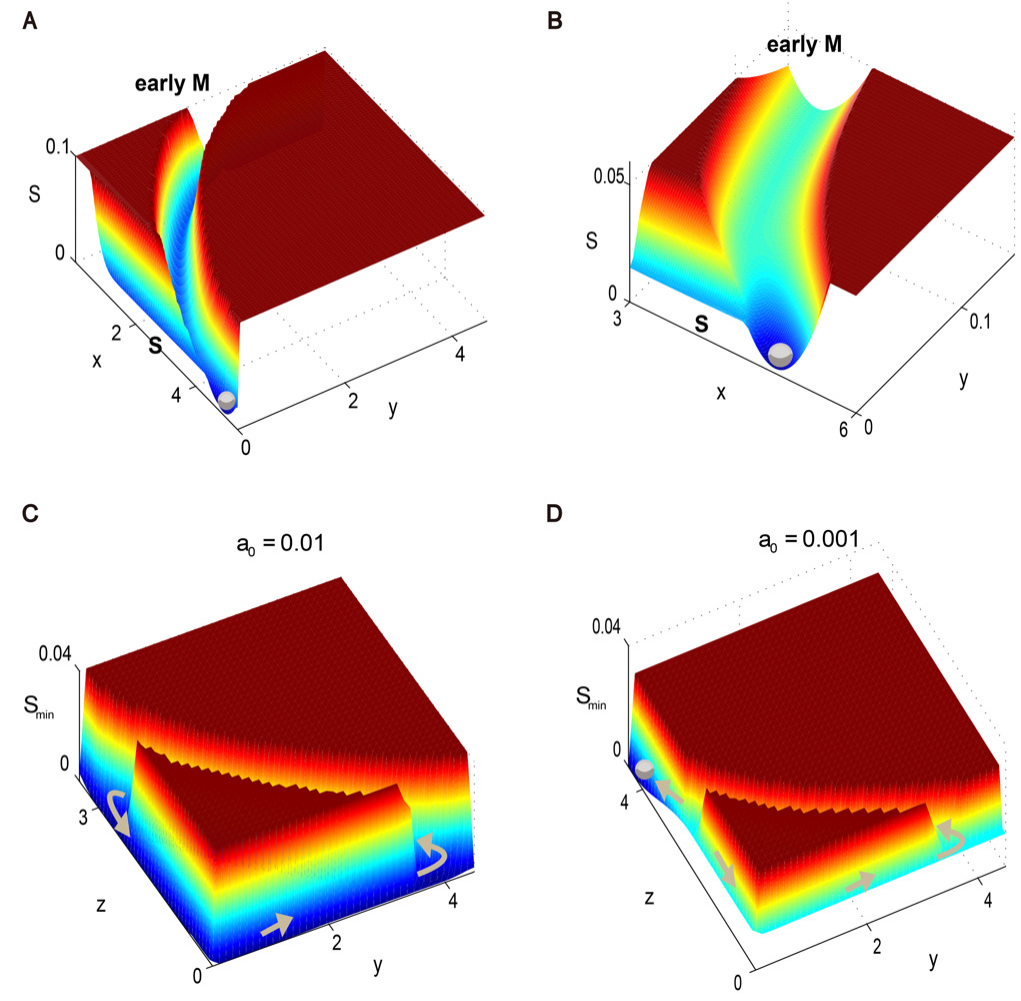
The energy landscape in response to external signals. (A) and (B) The energy landscape on the *z* = 0 plane, where *k*_*a*1_ = 0.0001 indicates that the DNA replication checkpoint is activated. (B) is the bottom right corner of (A). (C) and (D) The global energy landscape projected onto the *y*-*z* plane by choosing the potential minimum with respect to *x* for fixed *y* and *z*. The direction of the non-gradient force is shown as gray arrows. The energy landscape exhibits (C) limit cycles with sufficient nutrients and (D) excitable dynamics with insufficient nutrients. The “S” and “early M” in bold refer to S phase and early M phase respectively.

First, let us discuss the energy landscape’s response to DNA damage in the S phase of the yeast cell cycle. When there is DNA damage in the S phase, the DNA replication checkpoint is activated [38]. In the cell cycle process, the checkpoint mechanism ensures the completion of early events before the beginning of later events so as to maintain the progression order of the cell cycle [18]. We can decrease the parameter *k*_*a*1_ to 0.0001 to simulate this effect (See S1 Text for more details), with the resulting new energy landscape on the *z* = 0 plane shown in Fig. 4A and 4B. The results show that the flat canal around *P*_3_ now turns into a small pit, which will keep the system in the *P*_3_ state until the DNA damage is repaired. Similarly, *k*_*a*2_ = 0.0001 can be used to simulate the M phase checkpoint.

Secondly, the yeast cells will divide continuously when they are cultured in a rich medium [14, 26], and we can increase *a*_0_ to 0.01 to simulate this effect (see [26] and S1 Text). The resulting energy landscape is shown in Fig. 4C, where the global three-dimension energy landscape is projected onto the *y*-*z* plane by choosing the potential minimum with respect to *x* for fixed *y* and *z*. The results demonstrate that the system shifts from an excitable system to a stable limit cycle system through bifurcation (*a*_0_ = 0.0025) when nutrition is sufficient, and the stable G1 state disappears. As a comparison, we show a similar projected energy landscape with *a*_0_ = 0.001 in Fig. 4D, where the cell cycle process is an excitable system.

### Finite volume effect

The previous results are all based on the assumption that the system volume (or system size), i.e., the copy number of considered species, goes to infinity. In other words, we only studied the fluctuation effects for perturbations by noises that are small compared to the reaction rates ***b***(***x***) in Equation (1a). This is a reasonable assumption for most biological systems. However, if the system nearly stagnates at some transition areas where its reaction rates are so small that they are of the same magnitude as the noise, the previous picture does not hold. In this case, some special phenomena will appear that do not fall into our traditional analysis.

**Fig 5:**
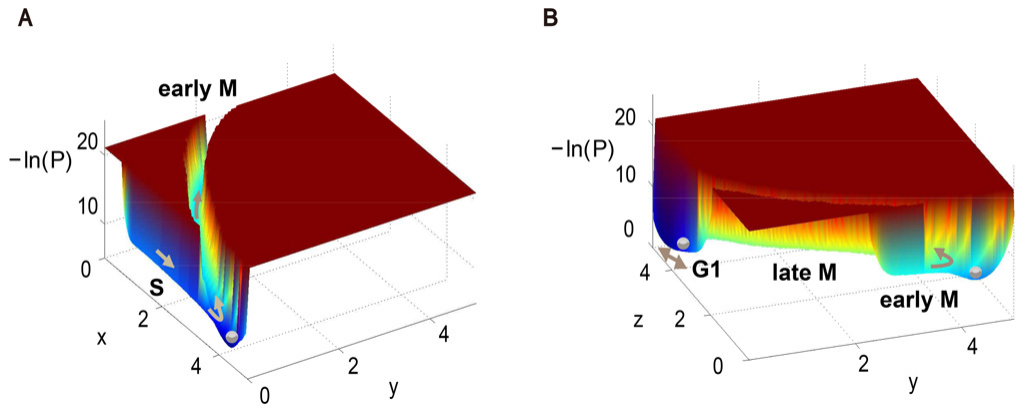
The energy landscape of the yeast cell cycle network with finite volume effect. (A) on the *x*-*y* plane with *z* = 0 and (B) on the *y*-*z* plane with *x* = 0. The moving direction of the system is shown as brown arrows. Here we use “−ln(*P*)” to define the energy landscape of the system, where *P* = *P*(***x***) is the stationary probability distribution of the system from simulation. The “G1”, “S”, “early M” and “late M” in bold refer to G1 phase, S phase, early M phase and late M phase respectively.

Here we use the classical Monte Carlo simulation method to study the finite volume effect. From Fig. 5 we can see that the landscape is generally unchanged except around two corners. The original flat landscape with long-lasting slow dynamical behavior near the checkpoints now turns into pits. In other words, these nearly degenerate points play the role of small potential wells along the canal. This is intuitively true because the stationary probability for each point on this unidirectional path is approximately determined by the speed with which the system passes across. Consequently the system transitions to having a multipeak probability distribution, where the additional peaks do not correspond to the stable points of ODEs in the usual case. These additional peaks are also found in the cell cycle process of mammalian cells [13]. This interesting finite volume effect is unique when our driving force strength is comparable to the noise strength in some places. The additional pits can be treated as a “fingerprint” for a multi-stage biological processes, in which the pits act as another kind of analogous checkpoint. We speculate that a similar phenomena also holds for the classical limit cycle dynamics.

### Intrinsic versus Extrinsic noise

So far we have only discussed the intrinsic fluctuations determined by the reaction network itself, and ignored the extrinsic influence from environments in which the noise strength is independent of the network structure [39–41]. However, we also investigated the energy landscape of our cell cycle model perturbed by extrinsic noise using a similar approach, and the result is shown in S2 Fig. Compared with the landscape obtained in Fig. 2 and 3, the general shape of the landscape is almost the same, but the width of the canal does not change as significantly as the one perturbed by intrinsic noise. This result coincides with the lower insensitive fluctuation strength in the extrinsic noise case (S2 Fig.). This point, which indicates our cell cycle model is more tolerant with respect to intrinsic noise, can be used to help distinguish between intrinsic randomness and any environmental perturbations of the system, especially for a multi-stage biological process that periodically changes its reaction rates in time.

## Discussion

**Fig 6:**
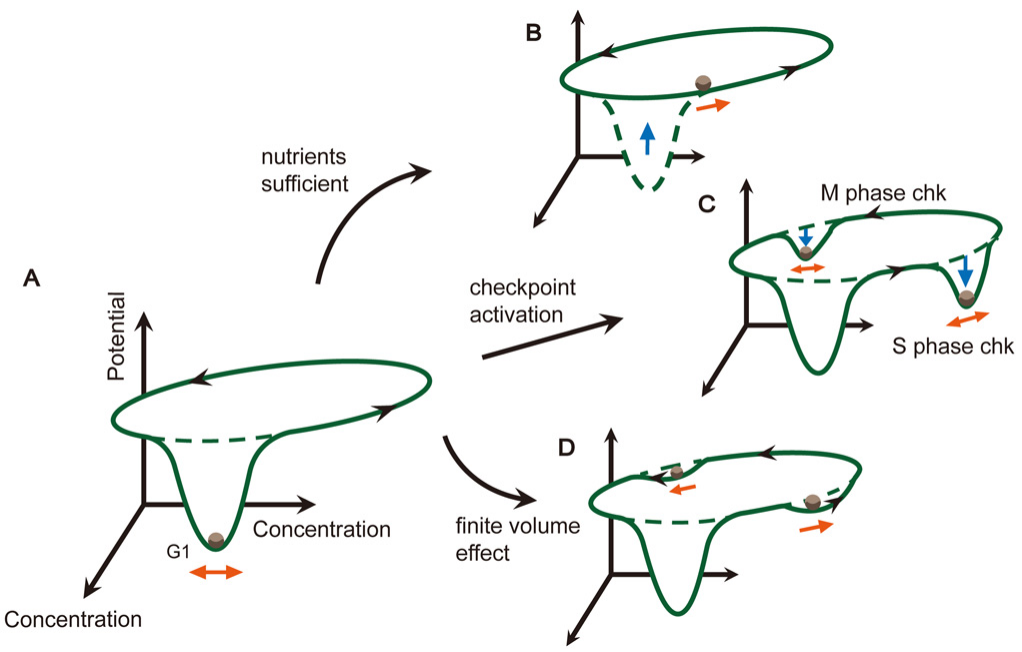
Summary of the schematic quasi-potential energy landscape for the yeast cell cycle network. (A) When nutrient availability is poor, the system has a global stable state shown as G1, and restrains the cell around this state. (B) When the amount of nutrients becomes sufficient, the system shifts to having a limit cycle, the cell is released and the cell cycle is activated. (C) When the S phase or M phase checkpoint mechanism is activated, a temporal stable state appears and holds the system there until the issue is resolved (the abbreviation of checkpoint is chk). (D) When taking finite volume effect into account, i.e. the noise strength is of the same magnitude as its reaction rates, the area with extremely slow rates in the cell cycle process lowers to form small pits that provide longer duration of stay when the system passes by. These new small pits play the role of metastable states. The black arrows represent the driving force on the landscape, the blue arrows illustrate the deformation of the landscape and the orange arrows show the movement of the system under noise perturbations.

We performed a careful study of the budding yeast cell cycle process from an energy-landscape point of view. The energy landscape of the budding yeast cell cycle is mainly comprised of two parts on a global scale: a deep pit that holds a cell in its G1 state when the environment is not suitable for division, and one unidirectional flat canal that performs a robust and accurate cell cycle progress once the system is excited (as summarized in Fig. 6A). When nutrients are sufficient, the original excitable system evolves into a stable limit cycle. Correspondingly, the deep pit lifts up, and the cell cycle can proceed efficiently without waiting in the G1 state (Fig. 6B). Once a cell meets any accident during its division, the corresponding cell cycle checkpoint is activated. In this case, the flat canal is dug to form an additional pit, and the system is held there until the accident is resolved (Fig. 6C). Furthermore, the super-slow dynamic stage on the canal corresponds to small pits if we take into account the finite volume effect. Those pits reduce the fluctuations from the cycle’s upstream processes and provides a longer stay duration when the system passes by (Fig. 6D).

Besides the global view, the energy landscape also contains massive details of the system. The unidirectional canal and the guardrail on both sides guarantee that each event occurs only once and in the right order. Although the global energy landscape defined through the stationary probability distribution does not contain the non-gradient effect, the local pseudo energy landscape we proposed clearly visualizes this unidirectionality brought by the non-gradient force. Now the strength of the non-gradient force along the canal is characterized by the steepness of the pseudo energy in the tangential direction. For a system perturbed by intrinsic noise, the fluctuation restriction ability in the transverse direction of the canal is highly related to the driving force strength at that point. However, for a system perturbed by extrinsic noise, this relationship is much weaker.

In our simplified model describing the essential dynamics of the yeast cell cycle process, we assumed the “domino” mechanism of cell cycle regulation, which is different from the previous Tyson’s model [42] and its landscape [9]. In our model, only the G1 phase cyclins are controlled by the cell mass, and the mitosis event in the M phase is triggered by the completion of the DNA replication event. This “domino” mechanism is also found in the cell cycle regulation of higher eukaryotic organisms [43]. With our model and new methods, we clearly identified the DNA replication and M phase checkpoints in the constructed energy landscape. This landscape can reshape itself in response to environmental nutrients (similar and consistent results in mammalian cell cycle [13]) and checkpoint signals adaptively. Furthermore, we proposed the concept local pseudo energy landscape to characterize the irreversibility of the dynamic path along the flat canal in the landscape. These points, to the authors’ knowledge, have not been revealed in the previous studies.

Due to the curse of dimensionality, we only performed our constructions for a three-node network model. When the problem is considered in higher dimensions, the computational cost increases exponentially. Even if this computational cost issue is resolved, however, determining how to save and exhibit such high-dimensional information remains a tough question. There is still a need to develop a systematic reduction method to analyze high dimensional problems.

Even with such limitations, we believe our meticulous study of the energy landscape of the simplified budding yeast cell cycle model is of general interest to those studying other complicated cell cycle dynamics. Most of the insights we gained studying this simple model are independent of the number of dimensions and the specific formulation of the model, and therefore will be valuable to other systems and studies.

## Supporting Information

S1 Text This file contains details that needed to understand the main body. It is arranged as follows: I. The three-node Budding Yeast Cell Cycle Model, II. Stochastic Model, III. Large Deviation Theory and the Hamiltonian, IV. Overview of the Construction of the Landscape, V. Introduction of the gMAM, VI. Construction of the Quasi-potential energy landscape, VII. Force Strength and Landscape Canal width, VIII. Analysis of the Non-gradient Force, IX. Signals Regulation in the Cell Cycle model, X. Extrinsic and Intrinsic Noise, XI. Parameters Sensitivity Analysis.
(PDF)

S1 Fig The regulatory network of cell-cycle process in budding yeast. (A) The regulatory network of key regulators in budding yeast cell-cycle process. It can be separated into G1/S, early M and late M modules, where the nodes represent cyclins, transcriptional factors and inhibitors, and the green and red lines represent activation (transcription) and the inhibition, respectively. (B) The essential network of yeast cell-cycle network, where *X*, *Y* and *Z* represent key regulators of the G1/S, early M and late M modules respectively, different modules are connected by activation and inhibition interactions. More details can be found in the main text [44].
(EPS)

S2 Fig The effects of extrinsic noise on the yeast cell-cycle network. (A) The quasi-potential energy landscape under extrinsic noise perturbation, the x-y plane where *z* = 0. (B) The ODE driving flux strength (black dashed line) in the ODE path and the fluctuation strength in the vertical direction of the ODE path perturbed by intrinsic noise (red solid line) and extrinsic noise (blue solid line). The letters ‘a’, ‘b’ and ‘c’ mark three points on the ODE trajectory with large force strength.
(EPS)

S3 Fig The inconformity between energy landscape and deterministic description of system. (A) The energy landscape in *x*-*y* plane with *z* = 0. (B) The pseudo energy landscape on *x*-*y* plane with *z* = 0. The red dashed boxes mark the energy barriers that contradict with the deterministic description of system.
(EPS)

S4 Fig Parameters sensitivity analysis of the system. Effects of parameter perturbations on global evolution trajectory (A-K) and the depth of energy well corresponding to the stable G1 state (L). In each subfigure, we either increase (blue solid line) or decrease (red dashed line) the value of a certain parameter by 20%. Subfigures from A to K, and the numbers on *x*-axis in subfigure (L) correspond to parameters: *j*_1_, *j*_2_, *j*_3_, *k*_1_, *k*_2_, *k*_3_, *k*_*i*_, *k*_*s*_, *k*_*a*1_, *k*_*a*2_, *a*_0_, respectively. (L) Δ*S* denotes the depth of energy well corresponding to the stable G1 state with wild-type parameters, and *δS* denotes the changes of depth after each parameter is increased or decreased.
(EPS)

S5 Fig Cell cycle with imperfect parameter values. The evolution trajectory (A) and the energy landscape on *x*-*y* plane with *z* = 0 (B) with imperfect parameter values: *j*_1_ = *j*_2_ = *j*_3_ = 0.5, *k*_1_ = *k*_2_ = *k*_3_ = 0.2, *k*_*i*_ = 5.0, *k*_*s*_ = 1.0, and *k*_*a*1_ = *k*_*a*2_ = 0.04.
(EPS)

S6 Fig The landscape on the *y*-*z* plane with *x* = 0 corresponds to the late M phase. In the second part of the cell cycle, the system evolves through the early M flat canal to the vertex *P*_4_ over a sufficient duration for the mitosis event. When the system passes through *P*_4_, *y* triggers the activation of the late M variable *z*, and the activated *z* begins to repress *y*. This is the transition from the early M phase to the late M phase. The “early M” and “late M” in bold refer to early M phase and late M phase respectively.
(EPS)
