# Supplementary material for: Energy Landscape Reveals That the Budding Yeast Cell Cycle Is a Robust and Adaptive Multi-stage Process
Source: PLoS Comput Biol. 2015 Mar 20;11(3):e1004156. doi: 10.1371/journal.pcbi.1004156 (PMC4368831; doi:10.1371/journal.pcbi.1004156)
Supplement: S1 Text — It is arranged as follows: I. The three-node Budding Yeast Cell Cycle Model, II. Stochastic Model, III. Large Deviation Theory and the Hamiltonian, IV. Overview of the Construction of the Landscape, V. Introduction of the gMAM, VI. Construction of the Quasi-potential energy landscape, VII. Force Strength and Landscape Canal width, VIII. Analysis of the Non-gradient Force, IX. Signals Regulation in the Cell Cycle model, X. Extrinsic and Intrinsic Noise, XI. Parameters Sensitivity Analysis. (PDF) [file pcbi.1004156.s001.pdf]

Text S1
Energy landscape reveals that the budding yeast cell cycle is a robust and adaptive multi-stage process

Supporting Information

Cheng Lv, Xiaoguang Li, Fangting Li, and Tiejun Li

Content

I. The three-node Budding Yeast Cell Cycle Model
II. Stochastic Model
III. Large Deviation Theory and the Hamiltonian
IV. Overview of the Construction of the Landscape
V. Introduction of the gMAM
 V-A. The Outer Loop
 V-B. Evaluating the Action
 V-C. The Inner Loop
VI. Construction of the Quasi-potential energy landscape
 VI-A. Landscape with One Stable State
 VI-B. Landscape with Two Stable States
 VI-C. Landscape with one stable limit cycle
 VI-D. Pseudo Landscape with the Non-gradient Force
 VI-E. Projecting the 3-D Landscape onto 2-D Plane
VII. Force Strength and Landscape Canal width
VIII. Analysis of the Non-gradient Force
IX. Signals Regulation in the Cell Cycle model
X. Extrinsic and Intrinsic Noise
XI. Parameters Sensitivity Analysis

I. THE THREE-NODE BUDDING YEAST CELL CYCLE MODEL

In this section, we first construct a key regulatory network for the budding yeast cell cycle process, then reduce it to a three-module network, and establish a coarse-grained three-node model to explore its essential architecture and function of cell cycle network rather than specific molecular components. We will utilize this three-node yeast cell cycle model to investigate the dynamic robustness of the system and adaptation to noise and outside signals. These coarse-graining approaches have been applied to study the designing principles of genetic regulatory networks [1–3].

Based on the yeast cell-cycle network (Figure 3-34 in [4]) with intra-S and spindle checkpoint [5], the budding yeast cell-cycle regulatory network can be constructed as shown in Fig. S1A, where the nodes represent cyclins, transcriptional factors and inhibitors, and the green and red lines represent activation (transcription) and the inhibition, respectively; and Cln2, Clb5 and Clb2 are used to represent Cln1,2, Clb5,6 and Clb1,2 respectively for simplification. We denoted it as the full cell cycle networks.

In the full cell cycle networks, the positive and negative feedback loops in the network play important role in governing yeast cell-cycle process. In the START point of G1 phase, the G1 cyclin Cln3 and CDK trigger the activation of transcriptional factors (TFs) SBF and MBF, SBF and MBF transcript the S phase cyclins Cln1,2 and Clb5,6. Cln1,2 and Clb5,6 will also activate SBF and MBF. This is the positive feedback in S phase [6]. Activated Cln3, Cln1,2 and Clb5,6 will also repress the activity of cell-cycle inhibitors, Whi5, Sic1, Cdh1/APC. S phase cyclins Cln1,2 and Clb5,6 form complex with CDK and start the events of daughter cell budding and DNA replication. At the end of S phase, the yeast cells finish the DNA replication event, then the DNA replication checkpoint mechanism is turned off. An unknown pathway will activate the activity of M phase TFs Mcm1/SFF. Mcm1/SFF transcribes the M phase cyclin Clb1,2 and Clb3,4, where Clb1,2 and Clb3,4 also activate Mcm1/SFF. This is the M phase positive feedbacks. The high level Clb1,2 will repress the activity of S phase TFs SBF and MBF. This is the negative feedback from early M phase to S phase. There is the metaphase/anaphase transition (M/A transition) in the M phase that spindle assemble and separation event is executing, and if there is any error the spindle checkpoint should be turned on to stop the consequent event only when the spindle assemble and separation is well finished. In the late M phase, the inhibitors Cdh1/APC and Cdc20/APC are activated to degrade the M phase cyclins Clb1,2 and Clb3,4, this is

the negative feedback from late M phase to early M phase. The positive feedbacks had been found between Pds1 and Cdc14 in spindle separation process [7], and complex regulations in mitotic exit process [8,9]. In the end of yeast cell-cycle, the inhibitor Whi5, Sic1 and Cdh1/APC are activated.

In Fig. S1A, the yeast cell-cycle regulatory network can be separated into G1/S, early M and late M modules. Based on this fact, a fundamental simplified 3-module network is constructed in Fig.S1B to focus on the interactions and regulations among the G1/S phase, early M phase and late M phase modules. In Fig.S1B, different modules are connected with activation and repression interactions, and x represents the concentration of activities of the key regulators in the excited G1/S phase, such as cyclins Cln2, Clb5 and transcriptional factors SBF and MBF. y represents the key regulators activities in early M phase, such as cyclins Clb2 and transcriptional factor Mcm1/SFF. And z represent the key inhibitors activities in late M/G1 phase, they are Cdh1, Cdc20 and Sic1.

The deterministic equations we used to model this three-node network are shown as below:

$$\frac{\mathrm{d}x}{\mathrm{d}t} = \frac{x^2}{j_1^2 + x^2} - k_1 x - xy + a_0, \tag{S1a}$$

$$\frac{\mathrm{d}y}{\mathrm{d}t} = \frac{y^2}{j_2^2 + y^2} - k_2 y - yz + k_{a1} x, \tag{S1b}$$

$$\frac{\mathrm{d}z}{\mathrm{d}t} = \frac{k_s z^2}{j_3^2 + z^2} - k_3 z - zx + k_{a2} y. \tag{S1c}$$

Parameter sets are searched to ensure the S phase and M phase event order and long duration time for both events. One typical set of parameters are $j_1 = j_2 = j_3 = 0.5,\ k_1 = k_2 = k_3 = 0.2,\ k_i = 5.0,\ k_s = 1.0,\ k_{a1} = k_{a2} = 0.001$. The corresponding evolution trajectories as the function of time and the trajectories in phase space are shown in Fig. 1B and C.

At the beginning of the cell-cycle process, in the G1 phase state, inhibitor z is in high level with low level of x and y, this is the P1 in the main text Figure 1C. The cell is in the resting G1 state. Once the system enters the cell-cycle process, it is excited G1 state and P2 in the main text Figure 1C, then the x wave is activated and represses the inhibitor z to lower level. In S phase the x wave is activated and dominant, in early M phase y wave is activated and dominant, and activated y represses x in turn. In late M and G1 phase z wave is triggered by y and increases to high level. We denote this as wave transition model of yeast cell-cycle.

More details and analysis of the three-node deterministic model concerning network reduction, model assumptions, formulation, and parameter setting can be found in a recent manuscript by one of the current authors (arXiv:1312.5204 [q-bio.MN]) [10].

II. STOCHASTIC MODEL

We model the cell cycle process with intrinsic noise as a Gillespie's jump process [11]. In this section, we illustrate how to construct a jump process based on Equation (S1).

We denote state as a vector $\boldsymbol{X} = (X, Y, Z)$, where each component represents the number of molecules for the corresponding specie. Then translate each term in (S1) into a chemical reaction. Taking equation (S1a) as an example, we have four associated reactions for the four terms. The state change vector for each reaction channel has the form $\boldsymbol{\nu}_1 = \boldsymbol{\nu}_4 = [1, 0, 0]$ and $\boldsymbol{\nu}_2 = \boldsymbol{\nu}_3 = [-1, 0, 0]$, which corresponds to the plus or minus sign in the equation. Once the j-th reaction fires, the state of the system $\boldsymbol{X}$ would be updated to $\boldsymbol{X} + \boldsymbol{\nu}_j$. The reaction propensity function is determined by each term and the volume size (or system size) V, where $\epsilon \equiv V^{-1}$ characterizes the magnitude of intrinsic fluctuations. In (S1a), the four propensity functions are

$$a_1(\boldsymbol{X}) = \frac{VX^2}{(j_1 V)^2 + X^2},\ a_2(\boldsymbol{X}) = k_1 X,\ a_3(\boldsymbol{X}) = \frac{XY}{V},\ a_4(\boldsymbol{X}) = a_0 V.$$

Equation (S1b) and (S1c) and be translated similarly. The propensity functions and state change vectors are listed in Table I. With these setup the stochastic reaction process $\boldsymbol{X}(t)$ can be established [11].

We choose these propensity functions because $a(\boldsymbol{X}) \sim O(V)$ when $\boldsymbol{X} \sim O(V)$. The concentration process $\boldsymbol{x}(t) \equiv \boldsymbol{X}(t)/V$ tends to deterministic process (S1) as V tends to infinity. To show this, we first write down the Kolmogorov backward equation for $u(t, \boldsymbol{X})$ defined as the expectation of any smooth function $f(\boldsymbol{X}(t))$ starting from $\boldsymbol{X}$ at time 0

$$\frac{\partial u}{\partial t} = \sum_j a_j(\boldsymbol{X})(u(t, \boldsymbol{X} + \boldsymbol{\nu}_j) - u(t, \boldsymbol{X})). \tag{S2}$$

TABLE I: Propensity functions and state change vectors

	Reaction 1	Reaction 2	Reaction 3	Reaction 4
State change vector	$[1,0,0]$	$[-1,0,0]$	$[-1,0,0]$	$[1,0,0]$
Propensity function	$\dfrac{VX^2}{(j_1V)^2+X^2}$	k_1X	$\dfrac{XY}{V}$	a_0V
	Reaction 5	Reaction 6	Reaction 7	Reaction 8
State change vector	$[0,1,0]$	$[0,-1,0]$	$[0,-1,0]$	$[0,1,0]$
Propensity function	$\dfrac{VY^2}{(j_2V)^2+Y^2}$	k_2Y	$\dfrac{YZ}{V}$	$k_{a1}X$
	Reaction 9	Reaction 10	Reaction 11	Reaction 12
State change vector	$[0,0,1]$	$[0,0,-1]$	$[0,0,-1]$	$[0,0,1]$
Propensity function	$\dfrac{Vk_sZ^2}{(j_3V)^2+Z^2}$	k_3Z	$\dfrac{ZX}{V}$	$k_{a2}Y$

where $u=u(t,\boldsymbol{X})$ is a smooth function defined on $\mathbb{R}\times\mathbb{N}^3$. The concentration variable $\boldsymbol{x}\equiv\boldsymbol{X}/V$ lives on the rescaled lattice $\mathbb{N}^3/V$. Denoting $\bar{a}_j(\boldsymbol{x})\equiv a_j(\boldsymbol{X})/V$, we rewrite equation (S2) in terms of $\boldsymbol{x}$ and $\bar{a}$:

$$\frac{\partial u}{\partial t}=V\sum_j \bar{a}_j(\boldsymbol{x})(u(t,\boldsymbol{x}+\boldsymbol{\nu}_j/V)-u(t,x)) \tag{S3}$$

$$=\sum_j \bar{a}_j\boldsymbol{\nu}_j(\boldsymbol{x})\frac{\partial u}{\partial x}+O(\frac{1}{V}). \tag{S4}$$

So when V tends to infinity, the $O(1/V)$ term vanishes and we have

$$\frac{\partial u}{\partial t}=\sum_j \bar{a}_j\boldsymbol{\nu}_j(\boldsymbol{x})\frac{\partial u}{\partial x}. \tag{S5}$$

This equation exactly corresponds to the backward equation of the deterministic process

$$\frac{\mathrm{d}\boldsymbol{x}}{\mathrm{d}t}=\sum_j \bar{a}_j\boldsymbol{\nu}_j, \tag{S6}$$

and this is exactly Equation (S1). In our case, $\bar{a}_1(\boldsymbol{x})=\frac{x^2}{j_1^2+x^2}, \bar{a}_2(\boldsymbol{x})=k_1x, \bar{a}_3(\boldsymbol{x})=xy$ and $\bar{a}_4(\boldsymbol{x})=a_0$.

III. LARGE DEVIATION THEORY AND THE HAMILTONIAN

In the presence of small random perturbations in dynamical systems, the behavior of the systems can not be described by the deterministic models all the time and the effect of the noise becomes ubiquitous. When the events with very little likelihood occurs, large deviation theory gives a rough estimate for the probability that the trajectory $X^\varepsilon(t), t\in[0,T], T<\infty$, of the random dynamical system lies in a small neighborhood around a given path $\psi\in C(0,T)$, where $C(0,T)$ denotes the space of all continuous functions mapping from $[0,T]$ into $\mathbb{R}^n$ [12]. The theory asserts that, for δ and ε sufficiently small,

$$\mathbb{P}_x\{\sup_{0\le t\le T}|X^\varepsilon(t)-\psi(t)|\le\delta\}\approx\exp(-\varepsilon^{-1}S_T(\psi)), \tag{S7}$$

where sup is short for supremum, which means the least upper bound of a subset. $\mathbb{P}_x$ denotes the probability conditioned on $X^\varepsilon(0)=x$ and assume $\psi(0)=x$. The action can be written as

$$S_T(\psi)=\begin{cases}\int_0^T L(\psi,\dot{\psi})dt & \text{if } \psi\in C(0,T) \text{ is absolutely continuous and the integral converges,}\\ +\infty & \text{otherwise,}\end{cases} \tag{S8}$$

where the Lagrangian $L(x,y)$ is given by

$$L(x,y) = \sup_{\theta\in\mathbb{R}^n} (\langle y,\theta\rangle - H(x,\theta)). \tag{S9}$$

Here $\langle\cdot,\cdot\rangle$ denotes the Euclidean scalar product in $\mathbb{R}^n$ and $H(x,\theta)$ is the Hamiltonian, x is the generalized coordinate, y is the time derivative of x and θ is the generalized momentum.

In our work, we mainly focus on two kinds of dynamical systems: diffusion process and Markov jump process. Here we give the Hamiltonian of each type. Readers may find more details in [13]. For a diffusion process on $\mathbb{R}^n$ with drift vector b and diffusion tensor $a = \sigma\sigma^T$, i.e.,

$$\mathrm{d}X^\varepsilon(t) = b(X^\varepsilon(t))\,\mathrm{d}t + \sqrt{\varepsilon}\sigma(X^\varepsilon(t))\,\mathrm{d}W(t). \tag{S10}$$

The Hamiltonian H and Lagrangian L are given by

$$H(x,\theta) = \langle b(x),\theta\rangle + \frac{1}{2}\langle\theta, a(x)\theta\rangle\,, \tag{S11}$$

$$L(x,y) = \frac{1}{2}\left\langle y-b(x), a^{-1}(x)(y-b(x))\right\rangle. \tag{S12}$$

As for a continuous-time Markov jump processes on $\varepsilon\mathbb{Z}^n$ with a generator Q defined for every test function $f : \mathbb{R}^n \to \mathbb{R}$ by

$$(Qf)(x) = \varepsilon^{-1}\sum_{j=1}^{N} a_j(x)\big(f(x+\varepsilon\nu_j) - f(x)\big), \tag{S13}$$

where $a_j : \mathbb{R}^n \to (0,+\infty), j = 1,\dots,N$, are the rates (or propensities) and $\nu_j \in \mathbb{Z}^n, j = 1,\dots,N$, are the state change (or stoichiometric) vectors. The Hamiltonian H for this type of dynamics is given by

$$H(x,\theta) = \sum_{j=1}^{N} a_j(x)(e^{\langle\theta,\nu_j\rangle} - 1), \tag{S14}$$

and L must be obtained via (S9)—in this case, no closed-form expression for L is available in general.

In our work, we use Eq. (S14) as our general Hamiltonian under intrinsic noise and Eq. (S11) for white noise which mimic the extrinsic noise with $a = id$. The corresponding Hamiltonian for these two cases are

$$\begin{aligned} H_{\text{int}}(x_1,x_2,x_3,\theta_1,\theta_2,\theta_3) = \;& \frac{x_1^2}{j_1^2+x_1^2}(e^{\theta_1}-1) + (k_1x_1 + x_1x_2)(e^{-\theta_1}-1) \\ &+(k_{a1}x_1 + \frac{x_2^2}{j_2^2+x_2^2})(e^{\theta_2}-1) + (k_2x_2 + x_2x_3)(e^{-\theta_2}-1) \\ &+(k_{a2}x_2 + \frac{x_3^2}{j_3^2+x_3^2})(e^{\theta_3}-1) + (k_3x_3 + k_ix_1x_3)(e^{-\theta_3}-1) \end{aligned} \tag{S15}$$

and

$$\begin{aligned} H_{\text{ext}}(x_1,x_2,x_3,\theta_1,\theta_2,\theta_3) = \;& \Big[\frac{x_1^2}{j_1^2+x_1^2} - (k_1x_1 + x_1x_2)\Big]\theta_1 + \frac{1}{2}\theta_1^2 \\ &+\Big[(k_{a1}x_1 + \frac{x_2^2}{j_2^2+x_2^2}) - (k_2x_2 + x_2x_3)\Big]\theta_2 + \frac{1}{2}\theta_2^2 \\ &+\Big[(k_{a2}x_2 + \frac{x_3^2}{j_3^2+x_3^2}) - (k_3x_3 + k_ix_1x_3)\Big]\theta_3 + \frac{1}{2}\theta_3^2. \end{aligned} \tag{S16}$$

The local quasi-potential with respect to a stable state $\boldsymbol{x}_s$, is defined by minimizing action functional (S8). More precisely,

$$S(\boldsymbol{x};\boldsymbol{x}_s) = \inf_{T>0}\inf_{\psi(0)=\boldsymbol{x}_s,\psi(T)=\boldsymbol{x}} S_T(\psi), \tag{S17}$$

where $S(\boldsymbol{x};\boldsymbol{x}_s)$ is the local quasi-potential with respect to $\boldsymbol{x}_s$, ψ is any connecting path satisfing $\psi(0) = \boldsymbol{x}_s$ and $\psi(T) = \boldsymbol{x}$, and $S_T(\psi)$ is an action functional of ψ. Generally, we will use the algorithm gMAM described below to

obtain this quasi-potential. However, there is a special case that can be calculated explicitly. Suppose the dynamics is simply a gradient system with a single-well potential driven by small noise, i.e.

$$\dot{\boldsymbol{x}} = -\nabla U(\boldsymbol{x}) + \sqrt{\epsilon}\dot{\boldsymbol{w}}, \tag{S18}$$

where ϵ is a small parameter, and $\dot{\boldsymbol{w}}$ is the standard temporal Gaussian white noise with $\mathbb{E}\dot{\boldsymbol{w}}(t) = 0$ and $\mathbb{E}\dot{\boldsymbol{w}}(s)\dot{\boldsymbol{w}}(t) = \delta(s-t)$. We assume that $U(\boldsymbol{x}) \geq 0$ and $U(x_s) = 0$ is the unique minimum of $U(\boldsymbol{x})$. As shown in equation (S12), choosing $a = I$, $b(x) = -\nabla U(x)$, we have have

$$S_T(\psi) = \frac{1}{2}\int_0^T \left|\dot{\psi} + \nabla U(\psi)\right|^2 dt. \tag{S19}$$

Here, we consider $\psi(0) = \boldsymbol{x}_s, \psi(T) = \boldsymbol{x}$. First we show that $S_T(\psi) \geq 2U(\boldsymbol{x})$ for all ψ. Because

$$\begin{aligned} S_T(\psi) &= \frac{1}{2}\int_0^T \left|\dot{\psi} + \nabla U(\psi)\right|^2 dt \\ &= \frac{1}{2}\int_0^T \left|\dot{\psi} - \nabla U(\psi)\right|^2 dt + 2\int_0^T \left\langle \dot{\psi}, \nabla U(\psi)\right\rangle dt \\ &\geq 2\int_0^T \left\langle \dot{\psi}, \nabla U(\psi)\right\rangle dt. \end{aligned}$$

The last integral can be computed directly.

$$2\int_0^T \left\langle \dot{\psi}, \nabla U(\psi)\right\rangle dt = 2\int_{\boldsymbol{x}_s}^{\boldsymbol{x}} dU(\psi) = 2U(\boldsymbol{x}) - 2U(\boldsymbol{x}_s) = 2U(\boldsymbol{x}).$$

So $S_T(\psi) \geq 2U(\boldsymbol{x})$. On the other hand, we can choose a special $\hat{\psi}$ and $T > 0$ such that $\dot{\hat{\psi}} = \nabla U(\hat{\psi})$ and $\hat{\psi}(T) = \boldsymbol{x}$. For this special $\hat{\psi}$,

$$S_T(\hat{\psi}) = 2\int_{\boldsymbol{x}_s}^{\boldsymbol{x}} \nabla U(\hat{\psi}) d\hat{\psi} = 2U(\boldsymbol{x}).$$

This equality means $S(\boldsymbol{x}; \boldsymbol{x}_s) = 2U(\boldsymbol{x})$. So $S(\boldsymbol{x}; \boldsymbol{x}_s)$ is a generalization of the usual potential concept $U(\boldsymbol{x})$.

Moreover, we can show that the stationary distribution $P(\boldsymbol{x})$ satisfies $S(\boldsymbol{x}; \boldsymbol{x}_s) = -\lim_{\epsilon\to 0} \epsilon \log P(\boldsymbol{x})$. Since $P(\boldsymbol{x})$ satisfies the Fokker-Planck equation

$$\frac{1}{2}\nabla^2 P(\boldsymbol{x}) + \nabla\cdot(P(\boldsymbol{x})\nabla U(\boldsymbol{x})) = 0. \tag{S20}$$

One can easily check that $P(\boldsymbol{x}) = \frac{1}{Z}e^{-\frac{2U(\boldsymbol{x})}{\epsilon}}$ is the solution of equation (S20), where Z is a normalization factor.

However, if the driving force is not a gradient force $\nabla U(\boldsymbol{x})$, the quasi-potential will not give the full information about the total force but its 'gradient part'. In a mathematical form, if the dynamic is

$$\dot{\boldsymbol{x}} = \boldsymbol{b}(\boldsymbol{x}) + \sqrt{\epsilon}\dot{\boldsymbol{w}}, \tag{S21}$$

where the driving force $\boldsymbol{b}(\boldsymbol{x})$ guarantees a decomposition $\boldsymbol{b}(\boldsymbol{x}) = -\nabla U(\boldsymbol{x}) + \boldsymbol{l}(\boldsymbol{x})$, $\langle\nabla U(\boldsymbol{x}), \boldsymbol{l}(\boldsymbol{x})\rangle = 0$. Now the action functional

$$\begin{aligned} S_T(\psi) &= \frac{1}{2}\int_0^T \left|\dot{\psi} - \boldsymbol{b}(\psi)\right|^2 dt = \frac{1}{2}\int_0^T \left|\dot{\psi} + \nabla U(\psi) - \boldsymbol{l}(\psi)\right|^2 dt \\ &= \frac{1}{2}\int_0^T \left|\dot{\psi} - \nabla U(\psi) - \boldsymbol{l}(\psi)\right|^2 dt + 2\int_0^T \left\langle \dot{\psi}, \nabla U(\psi)\right\rangle dt \\ &\geq 2\int_0^T \left\langle \dot{\psi}, \nabla U(\psi)\right\rangle dt = 2U(\boldsymbol{x}). \end{aligned}$$

So $S_T(\psi) \geq 2U(\boldsymbol{x})$ also holds. On the other hand, we can choose a special $\tilde{\psi}$ which satisfies

$$\dot{\tilde{\psi}} = \nabla U(\tilde{\psi}) + \boldsymbol{l}(\tilde{\psi}),$$

then

$$S_T(\tilde{\psi}) = 2\int_0^T \left\langle \dot{\tilde{\psi}}, \nabla U(x) \right\rangle dt = 2U(\boldsymbol{x}).$$

This identity shows that quasi-potential $S(\boldsymbol{x};\boldsymbol{x}_s)$ is the 'gradient part' of the drving force. The non-gradient part $\boldsymbol{l}(\boldsymbol{x})$, however, is not involved.

For a dynamical system with non-gradient driving force, the relation

$$\lim_{\epsilon\to 0} -\epsilon \log P(\boldsymbol{x}) = S(\boldsymbol{x};\boldsymbol{x}_s)$$

is also valid. However, the proof is much more difficult than the gradient case. Interested readers may refer to [12] for more details.

IV. OVERVIEW OF THE CONSTRUCTION OF THE LANDSCAPE

In this section, we briefly describe the procedure of constructing the energy landscape for the cell cycle model. This process can be easily extended to more general models.

First, we translate equation(S1) to a jump process by means that described in section II. Now we can write down the Hamiltonian of this process according to formula (S14). The result is given by equation (S15).

The construction of global quasi-potential is quite involved. In simple terms, the global quasi-potential $S(x)$ is a proper sticking of two local version $S(x;x_{s1})$ and $S(x;x_{s2})$, where x_{s1}, x_{s2} are two stable fixed points of equation (S1a). The definition of local potential can be found in section V. In order to compute the local potentials, we need the gMAM (geometric Minimum Action Method), which is described in detail in section IV.

We can summarize the procedure in flow chart Algorithm 1. One may follow this guidelines and substitute a particular ODEs in this flow chart to obtain its own landscape.

ALGORITHM 1: Guide of constructing global quasi-potential

INPUT: The deterministic ODE
OUTPUT: Global quasi-potential, 2-D version $S(x,y)$, 3-D version $S(x,y,z)$ and pseudo-potential $\tilde{S}(x,y)$ if needed

1: Translate ODE to a Markov jump process. The change vector of each reaction is determined by the sign of each term, the reaction rate function is the value of the term, as described before;
2: Write down Hamiltonian of the Markov jump process according to equation (S14);
3: Compute stable fixed points $\boldsymbol{x}_{s1}, \boldsymbol{x}_{s2}$ of ODE;
4: For every $\boldsymbol{x} = (x,y,z)$, compute local potential $S(\boldsymbol{x};\boldsymbol{x}_{s1})$ and $S(\boldsymbol{x};\boldsymbol{x}_{s2})$ by algorithm gMAM. Details of gMAM is fully explained in section IV;
5: Sticking $S(\boldsymbol{x};\boldsymbol{x}_{s1})$ and $S(\boldsymbol{x};\boldsymbol{x}_{s2})$ to global quasi-potential $S(\boldsymbol{x})$ following the guide lines in section V-A, V-B and V-C;
6: Project 3-D potential $S(x,y,z)$ to a 2-D version $S(x,y)$ following the method in section V-E;
7: If pseudo-potential $\tilde{S}(\boldsymbol{x})$ is needed, the way to obtain it is illustrated in section V-D.
RETURN:3-D quasi-potential $S(x,y,z)$, 2-D version $S(x,y)$ and Pseudo potential $\tilde{S}(x,y)$.

V. INTRODUCTION OF THE GMAM

For the ease of readers, we here give a brief synopsis of the geometric Minimum Action Method (gMAM). One can find more details from the reference [13].

The geometric minimum action method (gMAM) is a variant of the minimum action method (MAM) [14]. Both of them are going to minimize the action functional required to compute local quasi-potential $V(x_1,x_2)$ in Freidlin-Wentzell theory and finding the minimizer.

One can obtain the quasi-potential of a system by minimize its action functional

$$V(x_1,x_2) = \inf_{T>0} \inf_{\psi\in\bar{C}_{x_1}^{x_2}(0,T)} S_T(\psi), \tag{S22}$$

where $\bar{C}_{x_1}^{x_2}(0,T)$ denotes the space of all absolutely continuous functions $f:[0,T]\to\mathbb{R}^n$ such that $f(0)=x_1$ and $f(T)=x_2$. This is a double minimization problem. The key idea behind the gMAM is to reformulate the Freidlin-Wentzell action functional on the space of curves, thus eliminates the minimization problem to only the spacial dimension, instead of the double minimization on both spacial and temporal dimensions in MAM.

Now we skip the proof and go straight to the essence and algorithm of the gMAM. The key idea is to reformulate (S22) geometrically in terms of curves $\gamma = \{\varphi(\alpha) \mid \alpha \in [0,1]\}$, where $\varphi : [0,1] \to \mathbb{R}^n$ is an arbitrary parameterizations of the curve γ. The main result in this direction is that the quasi-potential (S22) can be expressed as

$$V(x_1, x_2) = \inf_{\varphi \in \bar{C}_{x_1}^{x_2}(0,1)} \hat{S}(\varphi) \quad \text{with } \hat{S}(\varphi) = \sup_{\vartheta:[0,1]\to\mathbb{R}^n, H(\varphi,\vartheta)\equiv 0} \int_0^1 \langle \varphi', \vartheta \rangle d\alpha \tag{S23}$$

$$= \int_0^1 \langle \varphi', \hat{\vartheta}(\varphi, \varphi') \rangle d\alpha \tag{S24}$$

$$= \int_0^1 \frac{L(\varphi, \lambda\varphi')}{\lambda} d\alpha, \quad \lambda = \lambda(\varphi, \varphi'). \tag{S25}$$

Here, the functions $\hat{\vartheta}(x,y)$ is implicitly defined for all $x \in D$ and $y \in \mathbb{R}^n \backslash \{0\}$ as the unique solution $\hat{\vartheta}, \lambda \in \mathbb{R}^n \times [0,\infty)$ of the system

$$H(x, \hat{\vartheta}) = 0, \quad H_\theta(x, \hat{\vartheta}) = \lambda y, \quad \lambda \geq 0. \tag{S26}$$

Since there is no close form of Lagrangian generally, the algorithm of gMAM can be expressed in two steps. The first is solve (S26) to have $\hat{\vartheta}$ and λ, we call it innerloop. The second it that assuming $\hat{\vartheta}$ and λ are known, design a steepest-decent algorithm to solve Euler-Lagrange equation, we call it outerloop. Of course, if explicit form of Lagrangian is available, innerloop step can be ignored.

A. The Outer Loop

The Euler-Lagrange equation of the variational problem (S23) is given by([13]):

$$\begin{cases} -\lambda^2\varphi'' + \lambda H_{\theta x}\varphi' - H_{\theta\theta}H_x - \lambda\lambda'\varphi' = 0 \\ \varphi(0) = x_1, \varphi(1) = x_2. \end{cases} \tag{S27}$$

where$\lambda = \frac{\langle H_\theta, \varphi' \rangle}{|\varphi'|^2}$, and $H_x, H_\theta, H_{\theta\theta}$ are evaluated at $(\varphi', \hat{\vartheta}(\varphi, \varphi'))$.

In the steepest-descent algorithm, we use τ to represent time and α still the curve parameter. First we discretize $\varphi(\tau, \alpha)$ both in τ and α; i.e., we define $\varphi_i^k = \varphi(k\Delta\tau, i\Delta\alpha), k \in \mathbb{N}_0, i = 0, \ldots, N$, where $\Delta\tau$ is the time step and $\Delta\alpha = 1/N$ if we discretize the curve into $N+1$ points. Then we discretize the initial condition $\varphi(0,\alpha)$ to obtain $\{\varphi_i^0\}_{i=0,\ldots,N}$ and, for $k \geq 0$, use a two-step method to update these points, which is described in Algorithm 2.

ALGORITHM 2: Outerloop of gMAM

INPUT: Current φ_i^k, start point x_1 and end point x_2;
OUTPUT: Path of next step φ_i^{k+1}

1: Compute ${\varphi'}_i^k = (\varphi_{i+1}^k - \varphi_{i-1}^k)/(2/N)$, and run innerloop to obtain $\hat{\vartheta}_i^k = \hat{\vartheta}(\varphi_i^k, {\varphi'}_i^k)$ for $i = 1, \ldots, N-1$,;
2: Compute $\lambda_i^k = \langle H_\theta(\varphi_i^k, \hat{\vartheta}_i^k), {\varphi'}_i^k \rangle / |{\varphi'}_i^k|^2$ for $i = 1, \ldots, N-1$. Set $\lambda_0^k = 3\lambda_1^k - 3\lambda_2^k + \lambda_3^k$, $\lambda_N^k = 3\lambda_{N-1}^k - 3\lambda_{N-2}^k + \lambda_{N-3}^k$;
3: Compute ${\lambda'}_i^k = (\lambda_{i+1}^k - \lambda_{i-1}^k)/(2/N)$ for $i = 1, \ldots, N-1$.;
4: **WHILE** some stopping criterion is not fulfilled **DO**
5: Let $\{\tilde{\varphi}_i\}_{i=0,\ldots,N}$ be the solution of the linear system

$$\begin{cases} \dfrac{\tilde{\varphi}_i - \varphi_i^k}{\Delta\tau} = (\lambda_i^k)^2 \dfrac{\tilde{\varphi}_{i+1} - 2\tilde{\varphi}_i + \tilde{\varphi}_{i-1}}{1/N^2} - \lambda_i^k H_{\theta x}{\varphi'}_i^k + H_{\theta\theta}H_x + \lambda_i^k {\lambda'}_i^k {\varphi'}_i^k, \quad i = 1, \ldots, N-1, \\ \tilde{\varphi}_0 = x_1, \\ \tilde{\varphi}_N = x_2, \end{cases} \tag{S28}$$

where $H_{\theta x}, H_{\theta\theta}$, and H_x are evaluated at $(\varphi_i^k, \hat{\vartheta}_i^k)$.
6: Interpolate a curve across $\{\tilde{\varphi}_i\}_{i=0,\ldots,N}$ and discretize this curve to find $\{\varphi_i^{k+1}\}_{i=0,\ldots,N}$ so that the prescribed constraint on the parameterizations of φ is satisfied.
7: **END WHILE**
RETURN: Path of next step $\varphi_i^{k+1}, i = 0, 1, \ldots N$.

B. Evaluating the Action

After the outer loop, one can easily compute the value of the action following Algorithm 3.

ALGORITHM 3: Evaluating action

INPUT: Most probable path $\{\varphi_i^k\}_{i=0,\dots,N}$, computed by outerloop;
OUTPUT: Action value of $S(x_2;x_1)$
1: Compute ${\varphi'}_i^k, \hat{\vartheta}_i^k$, and λ_i^k as in Step 1 of Algortihm 2 for every $i=0,\dots,N$.;
2: Action value is

$$\hat{S} = \frac{1}{N}\Big(\frac{3}{2}\langle {\varphi'}_i^k, \hat{\vartheta}_1^k\rangle + \sum_{i=2}^{N-2}\langle {\varphi'}_i^k, \hat{\vartheta}_i^k\rangle + \frac{3}{2}\langle {\varphi'}_{N-1}^k, \hat{\vartheta}_{N-1}^k\rangle\Big). \tag{S29}$$

RETURN:$S(x_2;x_1) = S$.

C. The Inner Loop (Computing $\hat{\vartheta}(\varphi,\varphi')$)

In order to compute $\hat{\vartheta}(\varphi,\varphi')$ from (S26), we use function $h(\cdot)$ to denote the strictly convex and twice-differentiable function $H(\varphi,\cdot)$. The quadratically convergent routine is as follows. Set $p=0$ initially, one can do innerloop according to Algortihm 4.

ALGORITHM 4: Innerloop of gMAM

INPUT: Current $\varphi_i^k, i=0,1,\dots N$;
OUTPUT: General momentum $\hat{\vartheta}_i^k = \hat{\vartheta}(\varphi_i^k, {\varphi'}_i^k)$, $i=1,\dots,N-1$,;
1: Give a proper guess of $\hat{\vartheta}_i^k$, set them $\hat{\vartheta}^0$
2: **WHILE** Convergence criterion is not fulfilled **DO**
3:

$$\hat{\vartheta}^{p+1} := \hat{\vartheta}^p + h_{\theta\theta}^{-1}\big(\tilde{\lambda}(\hat{\vartheta}^p)\varphi' - h_\theta\big) \quad \text{with } \tilde{\lambda}(\hat{\theta}^p) := \left(\frac{\langle h_\theta, h_{\theta\theta}^{-1}h_\theta\rangle - 2h}{\langle \varphi', h_{\theta\theta}^{-1}\varphi'\rangle}\right)_+^{1/2}, \tag{S30}$$

where $w_+^{1/2} = \sqrt{w}$ if $w \geq 0$ and $w_+^{1/2} = 0$ otherwise, and where h, h_θ, and $h_{\theta\theta}$ are evaluated at $\hat{\vartheta}^p$;
4: p=p+1;
5: **END WHILE**
RETURN:$\hat{\vartheta}^k = \hat{\vartheta}^p$

It is shown in [13] that this algorithm guarantees local quadratic convergence. So when applying this algorithm, we need a proper initial guess of ϑ. However, we usually have little knowledge about ϑ. If one find in a particular problem, innerloop does not converge, we suggest the following improvement.

Equation (S26) can be viewed as Lagrange multiplier method that solves the constrained optimization problem:

$$\begin{cases} \max\langle\vartheta,\varphi'\rangle \\ s.t. \quad h(\vartheta) = 0 \end{cases} \tag{S31}$$

So we can first apply a quadratic penalty method on this optimization problem. That is, we solve unconstrained problem

$$\min \frac{1}{2}\mu h^2(\vartheta) - \langle\vartheta,\varphi'\rangle \tag{S32}$$

where μ is a penalty factor. We can simply use Newton method with a proper line search to solve this problem. We know from [15] that Newton method with exact line search guarantees global convergence. Although this step may be a little expansive in computing, we need only a few step to offer the original innerloop a proper initial value. It is also easy to show that this strategy guarantees global convergence.

VI. CONSTRUCTION OF GLOBAL QUASI-POTENTIAL ENERGY LANDSCAPE

We describe the method to construct the global quasi-potential energy landscape below. In general, the global quasi-potential is a proper sticking of local quasi-potentials. For a dynamic system with several stable states, one can define local quasi-potential with respect to a stable state x_0 as

$$S(x; x_0) = \inf_{T>0} \inf_{\varphi, \varphi(0)=x_0, \varphi(T)=x} \int_0^T L(\varphi, \dot{\varphi}) dt. \tag{S33}$$

It is easy to see that one can compute local quasi-potential by applying gMAM algorithm, i.e. equation (S29). Choosing x_0 as initial point and x final point, gMAM will return the value of $S(x; x_0)$.

In our work, we mainly deal with dynamic systems with one or two stable states and dynamic system with a stable limit cycle. We will describe ways of constructing global quasi-potential of each case below. One may find a systematic strategy of sticking local quasi-potential in [12].

A. Landscape with One Stable State

For a system with only one stable state, such as an excitable system, the global quasi-potential is just local one. So the procedure of construction of the Quasi-potential energy landscape simply applying gMAM, which is shown in Algortihm 5.

ALGORITHM 5: Constructing landscape for system with one stable state

---

INPUT: The deterministic ODEs
OUTPUT: Quasi-potential landscape $S(x)$
1: Work out the stable points x_s from deterministic equations;
2: **REPEAT**
3: Set x_s as the starting point, with different ending point P_i, run the gMAM once and obtain $S(P_i) = S(P_i; x_s)$;
4: **UNTIL** All of the points P_i you concerned have been computed.
RETURN:$S(P)$

B. Landscape with Two Stable States

For a system have two metastable state looks like "stable-saddle-stable", such as when the checkpoint mechanism is activated, the process of reconstruction is described as follow.

First, we choose the two stable points (x_{s1}, x_{s2}) solved by the deterministic equations as our starting and ending point and obtain $S(x_{s2}; x_{s1})$ and $S(x_{s1}; x_{s2})$. Denote the difference $\Delta S = S(x_{s1}; x_{s2}) - S(x_{s2}; x_{s1})$. Then to obtain the value of $S(x)$ of each point P_i, we run the gMAM twice with x_{on} and x_{off} as initial point respectively and P_i the final point. Now we have two values of action $S(P_i; x_{s1})$ and $S(P_i; x_{x2})$ respectively. Freidlin-Wentzell theory shows that $S(P_i) = \min(S(P_i; x_{s2}) + \Delta S, S(P_i; x_{s1}))$. Repeat this procedure for every P_i to get the global quasi-potential. We summarize the steps of reconstruct global quasi-potential in Algortihm 6:

C. Landscape with one stable limit cycle

The procedure is almost the same as Case A, i.e. the system with one stable state. The difference is that the original starting point x_s (stable points) now turns into any point on the limit cycle.

D. Pseudo Landscape with the Non-gradient Force

To further explore the details of the flat canal, we came up with a different way of constructing the energy landscape. The main idea behind this method is to temporarily remove the global stable state from our model and only focus on the cycling, flat canal after the saddle point. Thus the landscape constructed this way is only a local landscape and does not reflect the global stationary probability distribution any longer.

ALGORITHM 6: Constructing landscape for system with two stable states

INPUT: The deterministic ODEs
OUTPUT: Quasi-potential landscape $S(x)$
1: Work out the stable points x_{s1} and x_{s2} from deterministic equations;
2: Run gMAM twice to get $S(x_{s2};x_{s1})$ and $S(x_{s1};x_{s2})$ and compute $\Delta S = S(x_{s1};x_{s2}) - S(x_{s2};x_{s1})$;
3: **REPEAT**
4: For every point P_i,

$$S(P_i) = \begin{cases} S(P_i;x_{s1}) & \text{if } S(P_i;x_{s1}) \leqslant S(P_i;x_{s2}) + \Delta S \\ S(P_i;x_{s2}) + \Delta S & \text{otherwise} \end{cases}$$

5: **UNTIL** All of the points P_i you concerned have been computed.
RETURN:$S(P)$

The main idea behind our framework is to compulsively let the system move from a low-action point to a high-action point.The gMAM algorithm will find the transition path of maximum likelihood and give the action value at the same time. However, if the transition path contains a part of ODE trajectory (we will call it a downhill part), the action cost of this part will be zero, not a negative value. At this time the real action difference between the starting and ending point may not be truly reflected, and we need to recompute the action for each cycling path in the optimal transition path. The algorithm to get pseudo potential is Algortihm 7.

ALGORITHM 7: Constructing pseudo landscape

INPUT: The deterministic ODEs
OUTPUT: Pseudo landscape $\tilde{S}(x)$
1: Work out the stable points x_s from deterministic equations;
2: **REPEAT**
3: For every point P_i, set x_s as the starting point, run the gMAM once, obtain $S_0(P_i;x_s)$ and the corresponding optimal transition path L_{i0};
4: Find the downhill part in L_{i0}, say, downhill parts are $(l_1,r_1),(l_2,r_2)\ldots(l_k,r_k)$. Set new starting point r_j, ending point l_j and compute out the corresponding action $S(l_1;r_1), S(l_2;r_2), \ldots S(l_k;r_k)$,
5: $\tilde{S}(P) = S_0(P_i;x_s) - (S(l_1;r_1) + S(l_2;r_2) + \cdots + S(l_k;r_k))$.
6: **UNTIL** All of the points P_i you concerned have been computed.
RETURN:$\tilde{S}(P)$

E. Projecting the 3-D Landscape onto 2-D Plane

When we are only interested in the 2-D configuration of the landscape, we need to reduce our 3-D landscape into a 2-D version. Suppose we have already had the 3-D global quasi-potential $S(x,y,z)$ and the stationary distribution of the three types of proteins is

$$P(x,y,z) \propto \exp(-KS(x,y,z)). \tag{S34}$$

Note that our 2-D version quasi-potential $S(x,y)$ must also follow

$$P(x,y) \propto \exp(-KS(x,y)), \tag{S35}$$

where $P(x,y)$ is the stationary distribution of two types of proteins. By definition, this distribution is given by

$$P(x,y) = \int P(x,y,z)dz \propto \int \exp(-KS(x,y,z))dz \propto \exp(-KS(x,y)). \tag{S36}$$

Therefore we know that there is a normalization factor Z that

$$S(x,y) = -K^{-1}\ln \int \exp(-KS(x,y,z))dz + K^{-1}\ln Z. \tag{S37}$$

As the volume of the system goes to infinity (noise level goes to infinitely small), K tends to infinity, now we have:

$$S(x,y) = \lim_{K\to+\infty} -K^{-1}\ln\int \exp(-KS(x,y,z))dz. \tag{S38}$$

Now we apply Laplace's approximation. This theorem (readers may refer to [16])states that if $g(t)$ attain its minimum at t_0, and

$$g(t) = g_0 + g_1(t-t_0) + o(t-t_0)$$

where g_0, g_1 are constant, then as $K \to +\infty$

$$\int_a^b e^{-Kg(t)}dt \sim 2\left(\frac{1}{Kg_1}\right)^2 \exp(-Kg_0).$$

Here we take $S(x,y,z)$ as g, denote $S_0 = \min_z S(x,y,z)$, (S38) implies

$$\begin{aligned} S(x,y) &= \lim_{K\to+\infty} -K^{-1}\ln\int \exp(-KS(x,y,z))dz \\ &= \lim_{K\to+\infty} -K^{-1}\ln\left(2(KS_1)^{-2}\exp(-KS_0)\right) \\ &= \lim_{K\to+\infty} -\frac{\ln 2 - 2\ln(KS_1)}{K} + S_0 \\ &= S_0 = \min_z S(x,y,z). \end{aligned} \tag{S39}$$

This result implies we can construct the 2-D quasi-potential by taking minimum of 3-D quasi-potential $S(x,y,z)$ with respect to z.

VII. FORCE STRENGTH AND LANDSCAPE CANAL WIDTH

In this section, the path we focus on is exactly the path depicted by the ODE Eqs. (1), which is also the cycling path and the flat canal on the quasi-potential energy landscape. From Eqs. (1), we define the "Force strength" as $\sqrt{(\frac{\mathrm{d}x}{\mathrm{d}t})^2 + (\frac{\mathrm{d}y}{\mathrm{d}t})^2 + (\frac{\mathrm{d}z}{\mathrm{d}t})^2}$, which is used to describe the strength of the driving force.

In order to measure the restricting strength of the canal in the landscape, we investigate the change of the landscape in the transverse direction of the canal, i.e. the cell cycle trajectory after the saddle node. Our analysis is based on the idea that we constrain the system in the transverse plane of any point '$\boldsymbol{x}_0$' in the ODE path and use the coefficient of variation to curve its fluctuation strength.

First we should find two orthogonal directions in the transverse plane of the ODE trajectory. We denote these two directions as $\boldsymbol{v}_1$ and $\boldsymbol{v}_2$ and the direction of ODE trajectory as $\boldsymbol{v}_0$. Notice that the stationary distribution $p(\boldsymbol{x}) \asymp \exp\{-KS(\boldsymbol{x})\}$, we can expand $S(\boldsymbol{x})$ in the vicinity of the fix point $\boldsymbol{x}_0$ up to second order thus get the Gaussian approximation

$$p(\boldsymbol{x}) \simeq \frac{1}{(2\pi)^1|\boldsymbol{\Sigma}|^{\frac{1}{2}}}\exp\Big\{-\frac{1}{2K}(\boldsymbol{x}(v_1,v_2))\boldsymbol{\Sigma}(\boldsymbol{x}(v_1,v_2))^T\Big\}. \tag{S40}$$

Here, $\boldsymbol{\Sigma} = (S_{ij})_{2\times 2}$, and $|\boldsymbol{\Sigma}|$ is the determinant of matrix $\boldsymbol{\Sigma}$. Eq. (S40) holds only in the vicinity of $\boldsymbol{x}_0$ with standard deviations $\sigma_{v_1} = (KS''_{v_2v_2})/|\boldsymbol{\Sigma}|)^{\frac{1}{2}}$ and $\sigma_{v_2} = (KS''_{v_1v_1})/|\boldsymbol{\Sigma}|)^{\frac{1}{2}}$. Then we diagonalize the σ matrix to find the maximum eigenvalue $\sigma_{\max}$. In Fig. S2B the canal width is the normalized $\lambda_{\max}$ for each point in the ODE trajectory.

VIII. ANALYSIS OF THE NON-GRADIENT FORCE

From the view of classic mechanics, the most probable cycling path is given by a Hamilton canonical equation:

$$\frac{\mathrm{d}\boldsymbol{x}}{\mathrm{d}t} = \nabla_{\boldsymbol{p}}H(\boldsymbol{x},\nabla S(\boldsymbol{x})) = \boldsymbol{F}(\boldsymbol{x}), \tag{S41}$$

$$\frac{\mathrm{d}\boldsymbol{p}}{\mathrm{d}t} = -\nabla_{\boldsymbol{x}}H(\boldsymbol{x},\boldsymbol{p}), \tag{S42}$$

where $S(x)$ is the quasi-potential and the canonical momentum $\boldsymbol{p} \equiv \nabla S(x)$. Based on the fact that $H(\boldsymbol{x}, 0) \equiv 0$, we have $\nabla_{\boldsymbol{x}} H \equiv 0$ when $\boldsymbol{p} = 0$. At saddle point, $\boldsymbol{p} = \nabla S(x) = 0$, so the cycling path, i.e. the path passed saddle point, satisfies $\boldsymbol{p} = \nabla S(x) \equiv 0$, and

$$\frac{\mathrm{d}\boldsymbol{x}}{\mathrm{d}t} = \nabla_{\boldsymbol{p}} H(\boldsymbol{x}, 0)).$$

Note that $\nabla_{\boldsymbol{p}} H(\boldsymbol{x}, 0))$ is nothing but the deterministic dynamic $\boldsymbol{b}(\boldsymbol{x})$, so the cycling path is exactly the ODE trajectory.

If the dynamic system is a gradient system $\dot{\boldsymbol{x}} = -\nabla V(\boldsymbol{x})$, the quasi-potential is simply $S(x) = 2V(x)$. So $\nabla S(x) = 0$ implies the cycling path is $\dot{\boldsymbol{x}} = 0$. So the cycling phenomena is typically caused by non-gradient force.

As for a general path, we use the case of extrinsic noise to show some heuristic insight. For a diffusion process

$$\mathrm{d}X_t^{\varepsilon} = b(X_t^{\varepsilon})\,\mathrm{d}t + \sqrt{\varepsilon}\,\mathrm{d}W_t,$$

Feidlin-Wentzell theory [12] tells us that the drift term $b(x)$ has a decomposition

$$b(x) = -\nabla S(x) + l(x),$$

where $S(x)$ is quasi-potential, $l(x)$ is perpendicular to $\nabla S(x)$, $\langle \nabla S(x), l(x) \rangle = 0$. The uphill path, by Hamilton canonical equaiotn(S41), is given by

$$\frac{\mathrm{d}\boldsymbol{x}}{\mathrm{d}t} = \nabla S(x) + l(x).$$

So it can be seen that the most probable dynamic is driven by two forces: the gradient force $\nabla S(x)$ and the non-gradient force $l(x)$. If $l(x) = 0$, the dynamic is a gradient system and is governed by gradient force only. In general, these two force together determine the dynamic of the system. When gradient force is 0 or small, the most probable dynamic is dominated by non-gradient force. Although for a Markov jump process, the drift does not guarantees such a decomposition, our analysis about the combination of gradient and non-gradient force is still helpful. This intuitive can provide a clear understanding of the most probable dynamic, except the two exceptions we will discuss below.

One is the energy barrier in the bottom right corner of Fig. 2B in the maintext, also the circled area in Fig. S3A. Given the set of parameters we used one can easily find out that in this area, it always has $\frac{\mathrm{dy}}{\mathrm{d}t} > 0$. While in the energy landscape it turns out to be an energy barrier between the S phase canal and the M phase canal. This arises the problems that we cannot always tell the movement of the system purely by the energy landscape which essentially reflects the stationary probability distribution of the system, also we cannot always compare the stationary probability of two adjacent points in the state space purely from the deterministic description of the system (Eqs. [1]). Another inconformity is shown in the circled area in Fig. S3B.

IX. SIGNALS REGULATION IN THE CELL CYCLE MODEL

To make sure the cell cycle can be regulated by the outer signals and be more robust to the emergencies during the process, in budding yeast, the cell cycle control system can also responds to the signals both from the environmental and the cell cycle it controls. In the main text we show the quasi-potential energy landscape under two types of signal, one is the nutrients in the environment and the other is the checkpoint signal from the cell itself.

According to [17], a central metabolite of glucose catabolism, acetyl-CoA, induces CLN3 transcription by promoting the acetylation of histones present in its regulatory region. Thus in our computing (S1), we set $a_0 = 0.001$ when the nutrient is not appropriate for dividing, and set $a_0 = 0.01$ when the nutrient is enough. In our model we also assume the other parameters are not affected by the nutrient level.

When the DNA replication during S phase goes wrong, the DNA replication checkpoint is activated. On the one hand cell activates a pathway that tries to solve the replication problem, on the other hand, cell holds the signal that blocks the activation effect the S-CDK has on G2-CDK. In our computing (S1), we use parameter k_{a1} to represent the activation effect that the S-CDK has on G2-CDK. Thus we set $k_{a1} = 0.001$ when the activation effect exists, and $k_{a1} = 0.0001$ when the activation effect is blocked.

X. EXTRINSIC AND INTRINSIC NOISE

It is well known that when the system size V is large enough, a Markov jump process can be well approximated by a chemical Langevin equation (CLE) [18]. Suppose a jump process described by equation (S13), when system size is

large enough, this process can be approximated by CLE

$$\frac{\mathrm{d}X}{dt} = \sum_{j=1}^{N} a_j(X)\boldsymbol{\nu}_j + \sqrt{\epsilon}\sum_{j=1}^{N}\sqrt{a_j(X)}\boldsymbol{\nu}_j \dot{W}_j, \tag{S43}$$

where $\dot{W}_j, j = 1, 2, \ldots N$ are independent Gaussian white noise. In this equation, the drift term is nothing but the deterministic flux. The diffusion term, which measures the strength of noise, is positively correlated with the strength of the drift. Therefore, when the cycling process is perturbed by intrinsic noise, the width of main canal is positively correlated to the strength of flux. The larger $|\boldsymbol{b}(x)|$ is, the wider the main canal is. This observation coincides with the numerical result shown in Fig. 3B in the main text.

On the other hand, if the cycling process is perturbed by extrinsic noise, the dynamic is

$$\frac{\mathrm{d}X}{dt} = b(X) + \sqrt{\varepsilon}\dot{W},$$

from which noise strength is independent of the propensity. Therefore the width of main canal is independent of the strength of the drift (see Fig S2).

XI. PARAMETERS SENSITIVITY ANALYSIS

In the main text, we do not give the analysis of parameters sensitivity. The word "robustness" in the title mainly refers to the robustness of the system under perturbation in state space. Here in Fig. S4 we changed each parameter value and see their effects on the global evolution trajectory and the depth of energy well indicating the stable G1 state. In Fig. S4 A to K, we demonstrate the global evolution trajectory when each parameter value is increased or decreased by 20%. We can see that the global trend of evolution trajectory does not change much under single parameter perturbation. In Fig. S4L, we show the relative change of the depth of energy well corresponding to the stable G1 state under the same parameters perturbation. We can see that the well depth is sensitive to four parameters: j_1(#1), k_1(#4), k_s(#8), and a_0(#11). All the parameters in Eq. 1a (j_1, k_1, a_0) are in the list. We suggest this sensitivity partly comes from the lack of redundancy of our simplified model.

In the main text, we changed the values of three parameters $\{a_0,\ k_{a1},\ k_{a2}\}$ to simulate the effects of external signals. To be specific, we increased a_0 to simulate the effect of additional nutrients, and decreased k_{a1} and k_{a2} to simulate the effects of the activation of checkpoints. When we decrease a_0 to 0, the global landscape would almost be the same with $a_0 = 0.001$. However, when we increase k_{a1} or k_{a2} significantly, the global picture would be evidently different. This has been discussed in detail in [10] and we briefly discussed it from the view of energy landscape. Figure S5 demonstrate the deterministic evolution trajectory and the energy landscape on x-y plane with $z = 0$ when k_{a1} and k_{a2} are increased from 0.001 to 0.04. We will refer to them as imperfect parameters [10]. From Fig. S5 we can see that the sharp corners that guarantee enough duration time of each cell-cycle state disappear, and the energy landscape transforms to a less constraining one, and have wider canals in general.

---

[1] Tsai TY, Choi YS, Ma W, Pomerening JR, Tang C, Ferrell JE Jr. (2008) Robust, tunable biological oscillations from interlinked positive and negative feedback loops. Science 321:126-129

[2] Ma W, Trusina A, El-Samad H, Lim W, Tang C (2009) Defining Network Topologies that Can Achieve Biochemical Adaptation. Cell 138: 760-773.

[3] Lim WA, Lee CM, Tang C (2013) Design principles of regulatory networks: searching for the molecular algorithms of the cell. Mol Cell 49: 202-12.

[4] Morgan D (2007) The cell cycle: principles of control. New Science Press (United Kingdom). p55.

[5] Li F, Long T, Lu Y, Ouyang Q and Tang C (2004) The yeast cell cycle is designed robustly. Proceedings of the National Academy of Sciences of the United States of America, 101(14):4781-4786.

[6] Skotheim JM, Di Talia S, Siggia ED and Cross FR (2008) Positive feedback of G1 cyclins ensures coherent cell cycle entry. Nature, 454(7202):291-296.

[7] Holt L, Krutchinsky A and Morgan D (2008) Positive feedback sharpens the anaphase switch. Nature 454(7202):353-357.

[8] Lopez-Aviles S, Kapuy O, Novak B and Uhlmann F (2009) Irreversibility of mitotic exit is the consequence of systems-level feedback. Nature 459(7246):592-595.

[9] Lu Y and Cross F (2010) Periodic Cyclin-Cdk Activity Entrains an Autonomous Cdc14 Release Oscillator. Cell 141(2):268-279.

[10] Li F, Hu M, Zhao B, Yan H, Wu B and Ouyang Q (2014) A globally attractive cycle driven by sequential bifurcations containing ghost effects in a 3-node yeast cell cycle model, arXiv:1312.5204[q-bio.MN].
[11] Gillespie DT (1997) Exact stochastic simulation of coupled chemical reactions. J. Phys. Chem., 81(25):2340-2361.
[12] Freidlin MI and Wentzell AD (1998) Random perturbations of dynamical systems, 2nd edition. Springer, New York.
[13] Heymann M and Vanden-Eijnden E (2008) The geometric minimum action method: a least action principle on the space of curves. Comm. Pure Appl. Math., 61(8):1052-1117.
[14] E W, Ren W and Vanden-Eijnden E (2004) Minimum action method for the study of rare events. Comm. Pure Appl. Math., 57(5):637-656.
[15] Nocedal J and Wright SJ (2006) Numerical Optimization, Springer, 2nd Edition.
[16] Holmes MH (1995) Introduction to Perturbation Methods, Springer-Verlag, New York.
[17] Shi L and Tu BP (2013) Acetyl-CoA induces transcription of the key G1 cyclin CLN3 to promote entry into the cell division cycle in Saccharomyces cerevisiae. *Proc Natl Acad Sci USA* 110(18): 7318-7323.
[18] Gillespie DT, The chemical Langevin equations, J. Chem. Phys. 113 (2000), 297-306.

FIG. S1: The regulatory network of cell-cycle process in budding yeast. (A) The regulatory network of key regulators in budding yeast cell-cycle process. It can be separated into G1/S, early M and late M modules, where the nodes represent cyclins, transcriptional factors and inhibitors, and the green and red lines represent activation (transcription) and the inhibition, respectively. (B) The essential network of yeast cell-cycle network, where X, Y and Z represent key regulators of the G1/S, early M and late M modules respectively, different modules are connected by activation and inhibition interactions. More details can be found in the main text[10].

FIG. S2: The effects of extrinsic noise on the yeast cell-cycle network. (A) The quasi-potential energy landscape under extrinsic noise perturbation, the x-y plane where $z = 0$. (B) The ODE driving flux strength (black dashed line) in the ODE path and the fluctuation strength in the vertical direction of the ODE path perturbed by intrinsic noise (red solid line) and extrinsic noise (blue solid line). The letters 'a', 'b' and 'c' mark three points on the ODE trajectory with large force strength.

FIG. S3: The inconformity between energy landscape and deterministic description of system. (A) The energy landscape in x-y plane with $z = 0$. (B) The pseudo energy landscape on x-y plane with $z = 0$. The red dashed boxes mark the energy barriers that contradict with the deterministic description of system.

FIG. S4: Effects of parameter perturbations on global evolution trajectory (A-K) and the depth of energy well corresponding to the stable G1 state (L). In each subfigure, we either increase (blue solid line) or decrease (red dashed line) the value of a certain parameter by 20%. Subfigures from A to K, and the numbers on x-axis in subfigure (L) correspond to parameters: j_1, j_2, j_3, k_1, k_2, k_3, k_i, k_s, k_{a1}, k_{a2}, a_0, respectively. (L) ΔS denotes the depth of energy well corresponding to the stable G1 state with wild-type parameters, and δS denotes the changes of depth after each parameter is increased or decreased.

FIG. S5: The evolution trajectory (A) and the energy landscape on x-y plane with $z = 0$ (B) with imperfect parameter values: $j_1 = j_2 = j_3 = 0.5$, $k_1 = k_2 = k_3 = 0.2$, $k_i = 5.0$, $k_s = 1.0$, and $k_{a1} = k_{a2} = 0.04$.

FIG. S6: The landscape on the y-z plane with x=0 corresponds to the late M phase. In the second part of the cell cycle, the system evolves through the early M flat canal to the vertex P_4 over a sufficient duration for the mitosis event. When the system passes through P_4, y triggers the activation of the late M variable z, and the activated z begins to repress y. This is the transition from the early M phase to the late M phase. The "early M" and "late M" in bold refer to early M phase and late M phase respectively.
